# Have We Selected for Higher Mesophyll Conductance in Domesticating Soybean?

**DOI:** 10.1111/pce.15206

**Published:** 2024-10-27

**Authors:** Elena A. Pelech, Samantha S. Stutz, Yu Wang, Edward B. Lochocki, Stephen P. Long

**Affiliations:** ^1^ Department of Plant Biology University of Illinois at Urbana‐Champaign Urbana Illinois USA; ^2^ Carl R Woese Institute for Genomic Biology University of Illinois at Urbana‐Champaign Urbana Illinois USA; ^3^ School of Life Sciences Nanjing University Nanjing China; ^4^ Department of Crop Sciences University of Illinois at Urbana‐Champaign Urbana Illinois USA

**Keywords:** crop domestication, crop improvement, food security, mesophyll conductance, photosynthetic CO_2_ assimilation, photosynthetic induction, soybean, water use efficiency

## Abstract

Soybean (*Glycine max*) is the single most important global source of vegetable protein. Yield improvements per unit land area are needed to avoid further expansion onto natural systems. Mesophyll conductance (*g*_*m*_) quantifies the ease with which CO_2_ can diffuse from the sub‐stomatal cavity to Rubisco. Increasing *g*_*m*_ is attractive since it increases photosynthesis without increasing water use. Most measurements of *g*_*m*_ have been made during steady‐state light saturated photosynthesis. In field crop canopies, light fluctuations are frequent and the speed with which *g*_*m*_ can increase following shade to sun transitions affects crop carbon gain. Is there variability in *g*_*m*_ within soybean germplasm? If so, indirect selection may have indirectly increased *g*_*m*_ during domestication and subsequent breeding for sustainability and yield. A modern elite cultivar (LD11) was compared with four ancestor accessions of *Glycine soja* from the assumed area of domestication by concurrent measurements of gas exchange and carbon isotope discrimination (∆^13^C). *g*_*m*_ was a significant limitation to soybean photosynthesis both at steady state and through light induction but was twice the value of the ancestors in LD11. This corresponded to a substantial increase in leaf photosynthetic CO_2_ uptake and water use efficiency.

## Summary statement

Mesophyll conductance (*g*_*m*_) describes the ease of CO_2_ transfer from the sub‐stomatal cavity to Rubisco, improvement would increase photosynthesis without additional water loss. *g*_*m*_ likely doubled in soybean domestication, suggesting variation within the germplasm for exploitation by direct selection.

## 1 Introduction

Soybean (*Glycine max* [L.] Merr.) is among the most important agricultural seed crops globally as the largest single source of vegetable protein and the second largest source of vegetable oils. The major areas of production are North and South America, and eastern Asia (Specht et al. 2014; Anderson et al. 2019). Improved agronomic practices and intensive breeding programmes have resulted in sustained soybean yield increases. However, these have not been sufficient to satisfy increasing demand which has resulted in expansion onto more natural land (Specht et al. 2014; Anderson et al. 2019; Williams et al. 2021). Increasing photosynthetic efficiency, especially if this can be achieved without more water, may be one way to supplement breeding efforts and achieve more yield within existing land of cultivation (Burgess et al. 2023; Long, Marshall‐Colon, and Zhu 2015; Murchie, Pinto, and Horton 2009). In an agricultural setting, crop canopy leaves consistently experience light intensity fluctuations due to changing solar angles, cloud cover, wind and intraspecific shading (Pearcy 1990; Long et al. 2022). Soybean is also a common understory legume crop in intercropping systems where such light fluctuations are intensified by interspecific shading (Adeniyan and Ayoola 2006; Kamara et al. 2019; Li et al. 2020; Mbah, Muoneke, and Okpara 2009; Pelech, Alexander, and Bernacchi 2021; Pelech et al. 2023). However, the response of photosynthesis to fluctuating light is not instant and the resulting loss may cost up to 13% of potential carbon assimilation in soybean (Wang et al. 2020). Exploring the factors limiting photosynthesis under both dynamic and steady‐state light conditions may aid a sustainable improvement of soybean productivity.

Leaf photosynthetic CO_2_ assimilation (*A*) shows a slow rise or induction on shade to sun transitions before reaching steady state. Both induction and steady‐state photosynthesis can each be limited by (1) conductance of CO_2_ from the air around the leaf to the site of assimilation in the chloroplast, (2) the maximum rate of ribulose 1,5‐biphosphate (RuBP) carboxylation (*V*_*c*max_) and (3) electron transport rate for the regeneration of RuBP (*J*). The CO_2_ diffusion path is characterised by two spatially sequential components, stomatal conductance (*g*_*s*_) and mesophyll conductance (*g*_*m*_). The path from the leaf boundary layer to the intercellular airspaces across the stomata defines *g*_*sw*_, whereas *g*_*m*_ in C_3_ crops is the gas to liquid phase CO_2_ diffusion path between leaf intercellular airspaces to Rubisco within the chloroplast stroma of the mesophyll cells (Flexas et al. 2008). Across studies and crop species, significant variations in the speed of stomatal opening during light induction exist (Acevedo‐Siaca et al. 2020; De Souza et al. 2020; Long et al. 2022), limiting photosynthetic rates between 10% and 15% (McAusland et al. 2016). On the other hand, the understanding of the limitation imposed by *g*_*m*_ to *A* during induction and steady state has rarely been evaluated due to greater difficulty of estimating *g*_*m*_, especially under dynamic light conditions (Leverett and Kromdijk 2024; Salesse‐Smith, Driever, and Clarke 2022).

The response of *g*_*m*_ to shade to sun transitions has been quantified using two techniques: (1) combined measurements of gas exchange and chlorophyll fluorescence or (2) simultaneous measurements of gas exchange with carbon isotope discrimination (∆^13^C). Kaiser et al. (2017) used the ‘Variable *J*’ method (Harley et al. 1992) with the former technique. The study refrained from quantifying the limitation to *A* during induction due to the assumptions and estimation bias with the Variable *J* method, but the steady‐state values compared well to other techniques (Bernacchi et al. 2002; Flexas et al. 2008; von Caemmerer and Evans 2015). Using the ∆^13^C technique has been suggested a more accurate method of estimating *g*_*m*_ due to the high sensitivity and specificity of tunable‐diode laser (TDL) absorption spectroscopy (Flexas et al. 2018; Leverett and Kromdijk 2024). A mechanistic model has been used to determine *g*_*m*_ under dynamic conditions that relates ∆^13^C to the multiple carbon isotope fractionation events. This utilizes the different speeds at which the two CO_2_ isotopologues (^12^CO_2_ and ^13^CO_2_) diffuse and/or are processed in biochemical reactions. This technique was used to measure *g*_*m*_ in Arabidopsis and tobacco under non‐photorespiratory conditions by Sakoda et al. (2021) who found that the limitation of *g*_*m*_ to *A* was the smallest compared to the limitations imposed by *g*_*sw*_, *V*_*c*max_ and *J* through induction. However, Liu et al. (2022) used the same technique in two *Arabidopsis* lines and found the relative limitation *g*_*m*_ imposed on *A* during induction was > 35%. Liu et al. (2022) also re‐analysed the limitation data presented in Sakoda et al. (2021) by time integration and found a > 20% *g*_*m*_ limitation on *A* during induction.

Whether *g*_*m*_ imposes a significant limitation to *A* during light induction and/or at steady state in soybean remains to be explored. Soybean was domesticated from *Glycine soja* [Siebold & Zucc] in China 6000–9000 years ago (Kim et al. 2012) which is a vining plant that would have escaped much shade compared to the considerable self‐shading and sun‐flecking that occurs in today's dense soybean crop canopies, which can have a leaf area index of over six (Dermody, Long, and DeLucia 2006). In selecting for higher yield or water use efficiency, indirect selection for higher *g*_*m*_ could be expected if there is variation in *g*_*m*_ within the germplasm. Determining whether variation exists is key to understanding if there is sufficient variation to allow for direct breeding selection for increased *g*_*m*_, and in turn increased productivity and water use efficiency (Specht et al. 2014; Anderson et al. 2019). This study tests the question of whether during domestication and subsequent breeding an inadvertent selection for increased *g*_*m*_ occurred, given its pivotal role in both crop photosynthetic efficiency and use of water. The hypotheses tested are: (1) *g*_*m*_ is a significant limitation to soybean photosynthesis both during light induction and at steady state and (2) domestication and selection have both increased *g*_*m*_, corresponding with increased leaf photosynthesis and water use efficiency.

## 2 Methods and Materials

### 2.1 Accession Selection

Ancestral soybean accessions *G. soja* [Siebold & Zucc] were selected from the U.S. National Plant Germplasm System (https://npgsweb.ars-grin.gov/gringlobal/search). The *G. soja* accessions were chosen from locations in the assumed regions of N.E. China where the germplasm, subsequently introduced into N. America, was domesticated (Liu et al. 2020). Four such accessions in maturity groups II through IV were selected (Table 2). The domesticated high‐yielding elite cultivar LD11‐2170 of *G. max* [L.] Merr was used for comparison.

### 2.2 Growth Conditions

**Figure 1:**
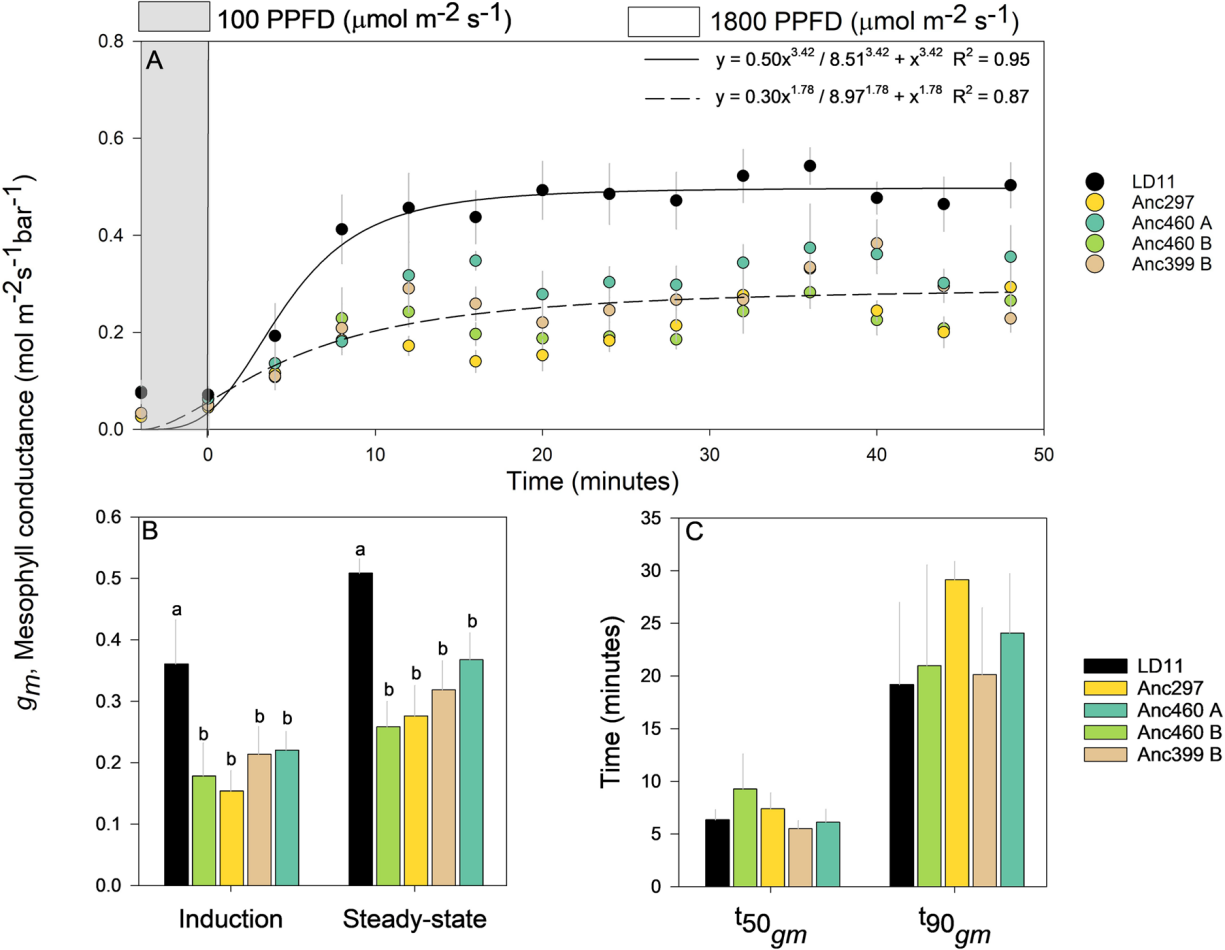
Comparisons among domesticated high‐yielding elite LD11 (*Glycine max* (L.) Merr) and four ancestor accessions (*Glycine soja* Siebold & Zucc) illustrated as (A) the average temporal response of mesophyll conductance (*g*_*m*_) after a transition in photosynthetic photon flux density (PPFD) from 100 (grey area) to 1800 (white area) where data points represent 4‐min moving averages, solid black line and dashed black line represent the fitted sigmoidal Hill regression of the elite and ancestors, respectively, (B) the average response of *g*_*m*_ during light induction and at steady state, and (C) the time taken for *g*_*m*_ to reach 50% ($t_{50g_{m}}$) and 90% ($t_{90g_{m}}$) of its steady‐state value. Light induction and steady state were defined as the initial and last 12 min (three 4‐min intervals) after an increase in light intensity, respectively. Different letters indicate significant differences (*p* < 0.1, repeated measures ANOVA, *n* = 12) and error bars indicate standard error.

To allow imbibition, the seed coats of ancestral accessions were cut, and the seed placed on wet paper towels for 1 week before sowing. Accessions were sown on 15 April 2022, at the University of Illinois Champaign‐Urbana in pots of 0.1 m depth filled with germination growing medium (Cultivation Nation Seventy Thirty Growing Media, Fox Farm, USA). Four to six seedlings of each accession were then transplanted after 7–10 days into 6‐L pots filled with the same growing medium as for germination but supplemented with 30 mL of slow‐release fertiliser (Osmocote Plus 15‐9‐12, ICL‐US). Once the ancestral accessions had established in the 6‐L pots, a 1 m wire tomato cage was inserted to each pot support tendril development (Supporting Information S1: Figure 1B). LD‐11‐2170 was grown alongside the ancestral accessions. Plants were watered twice daily. The average temperature of the greenhouse was 29.2°C with a 14‐h photoperiod.

### 2.3 Concurrent Measurements of Gas Exchange and Carbon Isotope Discrimination

Leaf gas exchange and photosynthetic carbon isotope discrimination were measured concurrently using an open‐gas exchange system (LI‐6800, LI‐COR Environmental, Lincoln, NE, USA) incorporating a clear‐top controlled environment small leaf chamber (6800‐17, LI‐COR Environmental, Lincoln, NE, USA) with the small LED light source (6800‐02, LI‐COR Environmental, Lincoln, NE, USA) enclosing 6 cm^2^ of leaf (Supporting Information S1: Figure 1A). On enclosure of the leaf, the settings were: chamber inlet [CO_2_] at 400 μmol mol^−1^, initial photosynthetic photon flux density (PPFD) 0 μmol m^−2^ s^−1^, flow rate 350 μmol s^−1^, air temperature at 25°C, vapour pressure deficit at 1.2 kPa and [O_2_] 1.97 kPa (2%). Once respiration was stable, six consecutive measurements were logged manually to measure dark respiration. Next, the auto programme was initiated where PPFD was set to 100 μmol m^−2^ s^−1^ for 24 min before increasing to 1800 μmol m^−2^ s^−1^ for 48 min. Measurements were recorded at 10 s intervals.

The gas‐exchange system was coupled to a TDL system (TDL; TGA200a, Campbell Scientific, Inc., Logan, UT, USA) for concurrent measurements of both [^12^CO_2_] and [^13^CO_2_], allowing estimation of δ^13^C (Bowling et al. 2003; Evans and Von Caemmerer 2013; Wang et al. 2022). The TDL was connected to the LI‐6800 reference air stream using the reference port on the back of the sensor head while the port on the front of the head supplied air from the leaf chamber (Jaikumar et al. 2021). CO_2_‐free air with 1.97 kPa [O_2_] and balance N_2_ was produced by mixing two gas streams using precision mass flow controllers (Omega Engineering Inc., Stamford, CT, USA) with a portion of the supply going to the gas exchange system while the remainder was used to calibrate and to correct for drift in the TDL (Jaikumar et al. 2021; Wang et al. 2022; Salesse‐Smith et al. 2024).

The TDL was calibrated using the concentration series method, described in detail in Wang et al. (2022). Briefly a 10% CO_2_ calibration cylinder was diluted in the N_2_/O_2_ stream to produce different [CO_2_] with the same isotopic composition (Tazoe et al. 2011; Ubierna et al. 2013; Wang et al. 2022). The measurement sequence consisted of eight gas streams: CO_2_‐free air, followed by three different [CO_2_] of the same isotopic signature, air from a calibration tank with a known [^12^CO_2_], [^13^CO_2_] and δ^13^C composition (NOAA Global Monitoring Laboratory, Boulder, CO, USA), the IRGA reference and leaf chamber air streams, and the IRGA reference again. As in Wang et al. (2022), each step had a duration of 20 s, except for the leaf chamber air, which had a duration of 600 s with a total cycle time of 740 s. Measurements were collected at 10 Hz and averaged over 10 s into a single data point. The first 10 s of each gas stream was excluded, except for the sample line which produced 59 data points each cycle according to Wang et al. (2022).

Before measurement, plants were kept in the dark overnight, and then transferred to a low PPFD of ca.10 µmol m^−2^ s^−1^. Between two and four 8–9‐week‐old plants of each accession in their vegetative growth phase were used for measurements. The youngest fully expanded trifoliate was selected. Given the vining architecture of the ancestral accessions, the youngest fully expanded trifoliate not overlapped by a neighbouring trifoliate or tendril was chosen. In some cases, the tendril with the selected trifoliate had to be disentangled to reach the leaf chamber (Supporting Information S1: Figure 1B).

### 2.4 Calculations of photosynthetic discrimination (Δ^13^C_obs_) and mesophyll conductance (*g*_*m*_)

On‐line photosynthetic discrimination (Δ^13^C_obs_) was calculated according to Evans et al. 1986:

(1) $$\Delta^{13}C_{\mathrm{obs}}=\frac{1000\xi \;(\delta^{13}C_{\mathrm{samp}}-\delta^{13}C_{\mathrm{ref}})}{1000+\delta^{13}C_{\mathrm{samp}}-\xi (\delta^{13}C_{\mathrm{samp}}-\delta^{13}C_{\mathrm{ref}})},$$

where δ^13^C_samp_ and δ^13^C_ref_ are the carbon isotope compositions of the leaf chamber and reference air in the LI‐6800 and *ξ* is

(2) $$\xi =\frac{C_{\mathrm{ref}}}{C_{\mathrm{ref}}-C_{\mathrm{samp}}},$$

where *C*_ref_ and *C*_samp_ are the CO_2_ concentrations of dry air entering and exiting the leaf chamber, respectively, measured by the TDL. A full list of symbols can be found in Table 1.

**Table 1:** Summary abbreviations and their definitions and units.

Variable	Definition	Units	Notes
*a*_*s*_	Fractionation across the stomata	‰	4.4
*a*_*b*_	Fractionation across the boundary layer	‰	2.9
*a*_*i*_	Fractionation factor for dissolution and diffusion through water	‰	1.8
*a*′	Combined fractionation factor through the leaf boundary layer and stomata	‰	Equation (5)
*A*	Net CO_2_ assimilation rate	μmol m^−2^ s^−1^	
*A*_*c*_	Rubisco‐limited net CO_2_ assimilation rate	μmol m^−2^ s^−1^	Equation (15)
*A*_*j*_	RuBP‐regeneration‐limited net CO_2_ assimilation rate	μmol m^−2^ s^−1^	Equation (16)
${A|}_{g_{sc}=\infty }$	Modelled assimilation rate expected to occur if stomatal conductance to CO_2_ diffusion were infinite	μmol m^−2^ s^−1^	Equation (11)
${A|}_{g_{m}=\infty }$	Modelled assimilation rate expected to occur if mesophyll conductance to CO_2_ diffusion were infinite	μmol m^−2^ s^−1^	Equation (12)
*b*	Fractionation associated with Rubisco carboxylation	‰	29 (Roeske and O'Leary 1984)
*C*_*a*_	Ambient CO_2_ partial pressure	µmol mol^−1^	
*C*_*c*_	Chloroplastic CO_2_ partial pressure/mole fraction	µmol mol^−1^	
${C_{C}|}_{g_{sc}=\infty }$	Value of $C_{C}$ expected to occur if stomatal conductance to CO_2_ diffusion were infinite	Pa/µmol^−1^	Equation (13)
${C_{C}|}_{g_{m}=\infty }$	Value of $C_{C}$ expected to occur if mesophyll conductance to CO_2_ diffusion were infinite	Pa/µmol^−1^	Equation (14)
*C*_*i*_	Intercellular CO_2_ partial pressure/mole fraction	µmol mol^−1^	
*C*_*s*_	CO_2_ partial pressure at the leaf surface	µmol mol^−1^	
*e*	Respiratory fractionation during decarboxylation (respiratory fractionation)	‰	0 (Ubierna et al. 2013)
*e*′	Fractionation during decarboxylation including measurement artefacts	‰	Equation (6)
*E*	Transpiration rate	mol H_2_O m^−2^ s^−1^	
*f*	Fractionation during photorespiration	‰	11.6 in this study
*g*_*m*_	Mesophyll conductance	μmol m^−2^ s^−1^ Pa or bar^−1^	Equation (3)
*g*_*sc*_	Stomatal conductance to CO_2_ diffusion; $g_{\mathrm{sc}}=g_{\mathrm{sw}}/1.6$	mol m^−2^ s^−1^	
*g*_*sw*_	Stomatal conductance to water vapour	mol m^−2^ s^−1^	
*g* ^*t*^_*ac*_	Total conductance to CO_2_ diffusion between boundary layer and stomatal conductance	mol total m^−2^ s^−1^	
*J*	Light‐dependent RuBP regeneration rate	μmol m^−2^ s^−1^	Equation (18)
$l_{s}^{\mathrm{Warren}}$	Relative limiting factor due to stomatal conductance in the Warren et al. (2003) framework	%	Equation (11)
$l_{m}^{\mathrm{Warren}}$	Relative limiting factor due to mesophyll conductance in the Warren et al. (2003) framework	%	Equation (12)
*O*	O_2_ partial pressure	Pa	
PPFD	Photosynthetic Photon Flux Density	µmol m^−2^ s^−1^	
*R*_*d*_	Mitochondrial respiration in the light	μmol CO_2_ m^−2^ s^−1^	
S_c/o_	Rubisco specificity factor	mol CO_2_/mol O_2_	
*t*	Ternary effect	‰	Equation (4)
$t_{50g_{m}}$	time taken for mesophyll conductance to reach 50% of its steady‐state value	minutes	
$t_{90g_{m}}$	time taken for mesophyll conductance to reach 90% of its steady‐state value	minutes	
*V*_*c*max_	Maximum Rubisco carboxylation rate	μmol m^−2^ s^−1^	Equation ((15), (17))
WUEi	Intrinsic water‐use efficiency	mol CO_2_ mol^−1^ H_2_O	*A*/*g*_sw_
Δ^13^C_obs_	Photosynthetic discrimination	‰	Equation (1)
Δ_*e*_	Fraction associated with respiration	‰	Equation (8)
Δ_*f*_	Fractionation associated with photorespiration	‰	Equation (9)
Δ_*i*_	Fractionation if *C*_i_ = *C*_c_ in the absence of any respiratory fraction	‰	Equation (7)
Γ*	CO_2_ compensation point in the absence of mitochondrial respiration in the light	Pa or bar	Equation (10)
ξ	Ratio of ^12^CO_2_ mole fraction in the dry air coming into the gas‐exchange cuvette over the difference in ^12^CO_2_ mole fractions of air in and out of the cuvette	unitless	Equation (2)

**Table 2:** Summary of soybean accession information.

Species	Accession	Maturity group	Country of origin
*Glycine soja* Siebold & Zucc	Anc297 (*PI 407297*)^a^	II	Liaoning Sheng, China
	Anc460 A (*PI 483460 A*)^a^	III	Liaoning Sheng, China
	Anc460 B (*PI 483460 B*)^a^	III	Liaoning Sheng, China
	Anc399 B (*PI 468399 B*)^a^	IV	Shandong Sheng, China
*Glycine max* (L.) Merr	LD11 (LD11‐2170)^b^	III	Illinois, United States

^a^ U.S. National Plant Germplasm System (https://npgsweb.ars-grin.gov/gringlobal/search).

^b^ Cai and Brock (2021).

Mesophyll conductance was estimated according to Evans and Von Caemmerer (2013), accounting for the ternary effect (Farquhar and Cernusak 2012):

(3) $$g_{m}=\frac{\frac{1+t}{1-t}\left(b-a_{i}-\frac{eR_{d}}{\left(A+R_{d}\right)}\right)\frac{A}{C_{a}}}{\Delta_{i}-\Delta_{o}-\Delta_{e}-\Delta_{f}},$$

where *b* is fractionation associated with Rubisco carboxylation (29‰, Roeske and O'Leary 1984), *a*_*i*_ (1.8‰) denotes the fractionation factor for dissolution and diffusion through water, *e* denotes the apparent fractionation factor associated with decarboxylation, *R*_*d*_ is the rate of mitochondrial respiration in the light (assumed to be equal to respiration in the dark) and *A* is the rate of net leaf CO_2_ assimilation. Carbon dioxide diffusion from the boundary layer to the sub‐stomatal cavity is affected by collisions of the isotopologues of CO_2_ with air and with water vapour, a process referred to as the ternary effect. Without accounting for this, conductance would be overestimated. Following Farquhar and Cernusak (2012) the ternary correction factor (*t*) is obtained as

(4) $$t=\frac{\left(1+a\prime \right)E}{2g_{\mathrm{ac}}^{t}},$$

where *E* is the rate of transpiration, *g* ^*t*^_*ac*_ is the total conductance to CO_2_ diffusion in the gas phase including boundary layer and stomatal conductance (von Caemmerer and Farquhar 1981) and *a*′ denotes the combined fractionation factor through the leaf boundary layer and stomata:

(5) $$a^{\prime }=\frac{a_{b}\left(C_{a}-C_{s}\right)+a_{s}(C_{s}-C_{i})}{C_{a}-C_{i}},$$

where *C*_*a*_, *C*_*s*_ and *C*_*i*_ are the ambient, leaf surface and intercellular CO_2_ partial pressures, *a*_*b*_ (2.9‰) is the fractionation occurring through diffusion in the boundary layer and *a*_*s*_ (4.4‰) is the fractionation due to diffusion in the air (Evans et al. 1986).

The apparent fractionation during decarboxylation includes two terms, such that *e* = *e*′ + *e*_0_. The first term *e*′ represents the influence of measurement artifacts, defined as

(6) $$e^{\prime }=\delta^{13}C_{\mathrm{ref}}-\delta^{13}C_{g\mathrm{atm}}$$

while *e*_0_ is the respiratory fractionation during decarboxylation, taken to be 0‰ in this study (Evans and Von Caemmerer 2013; Ubierna et al. 2013). δ^13^C_ref_ is the isotopic signature of the CO_2_ entering the LI‐6800 reference and δ^13^C_*g*atm_ (−8‰) is the isotopic compositions of the CO_2_ where the plants are grown. Δ_*i*_ is the discriminations that would occur if *C*_*i*_ = *C*_*c*_ in the absence of any respiratory fractionation (*e* = 0):

(7) $$\Delta_{i}=\frac{1}{\left(1-t\right)}a^{\prime }+\frac{1}{\left(1-t\right)}(\left(1+t\right)b-a^{\prime })\frac{C_{i}}{C_{a}}$$

Δ_*e*_ is the fractionation associated with respiration:

(8) $$\Delta_{e}=\frac{1+t}{1-t}\left(\frac{eR_{d}}{\left(A+R_{d}\right)C_{a}}(C_{i}-\Gamma^{*})\right),$$

$\Delta_{f}$ is the fractionation associated with photorespiration:

(9) $$\Delta_{f}=\frac{1+t}{1-t}\left(f\frac{\Gamma^{*}}{C_{a}}\right),$$

where *f* is the photorespiratory fractionation factor assumed to be 11.2‰ (Lanigan et al. 2008) and Δ_*f*_ is linearly related to O_2_ concentration via the CO_2_ compensation point in the absence of respiration ($\Gamma^{*}$):

(10) $$\Gamma^{*}=\frac{O}{2S_{c/o}},$$

where *O* is the [O_2_] in partial pressure and S_*c*/*o*_ is the Rubisco specificity factor (von Caemmerer, 2000).

### 2.5 Calculations of Limiting Factors

Dimensionless factors representing the limitations placed on the photosynthetic CO_2_ assimilation rate by CO_2_ diffusion or biochemistry were calculated using two different frameworks (Warren et al. 2003; Grassi and Magnani 2005) using the *calculate_c3_limitations_warren* and *calculate_c3_limitations_grassi* functions from the *PhotoGEA* R package (Lochocki 2024). Here, we describe the Warren et al. (2003) framework, the Grassi and Magnani (2005) framework can be found in Supporting Information S1: Methods.

Limiting factors during induction were calculated using the 4‐min averages of *A*, *g*_*sw*_, *g*_*m*_, *C*_*i*_ and *C*_*c*_, as described in detail below. The Warren et al. (2003) framework defines limiting factors due to stomatal and mesophyll conductance, which we refer to here as $l_{s}^{\mathrm{Warren}}$ and $l_{m}^{\mathrm{Warren}}$:

(11) $$l_{s}^{\mathrm{Warren}}=\frac{{A|}_{g_{sc}=\infty }-A_{n}}{{A|}_{g_{sc}=\infty }},$$

(12) $$l_{m}^{\mathrm{Warren}}=\frac{{A|}_{g_{m}=\infty }-A_{n}}{{A|}_{g_{m}=\infty }},$$

where *A* is the measured assimilation rate and ${A|}_{g_{sc}=\infty }$ and ${A|}_{g_{m}=\infty }$ are modelled assimilation rates expected to occur if *g*_*sc*_ (stomatal conductance to CO_2_ diffusion *g*_*sc*_ = *g*_*sw*_/1.6) or g_*m*_ were infinite, respectively. These modelled rates can be calculated by first defining corresponding values of *C*_*c*_ using *C*_*c*_ = *C*_*a*_ *– ∆C*_*s*_−*∆C*_*m*_, where *∆C*_*s*_ = *C*_*a*_ *− C*_*i*_ and *∆C*_*m*_ = *C*_*i*_ *− C*_*c*_ are the [CO_2_] drawdowns across the stomata and mesophyll, respectively, and *C*_*a*_, *C*_*i*_ and *C*_*c*_ are the measured ambient, intercellular and chloroplast [CO_2_], respectively. If conductance across a barrier (stomata or mesophyll) is infinite, the drawdown across it is zero:

(13) $${C_{C}|}_{g_{sc}=\infty }=C_{a}-0-\left(C_{i}-C_{c}\right)=C_{a}-C_{i}+C_{c},$$

(14) $${C_{C}|}_{g_{m}=\infty }=C_{a}-\left(C_{a}-C_{i}\right)-0=C_{i},$$

where ${C_{C}|}_{g_{sc}=\infty }$ and ${C_{C}|}_{g_{m}=\infty }$ are chloroplast [CO_2_] that expected to occur if *g*_*sc*_ or *g*_*m*_ were infinite, respectively. With these, it is possible to calculate the modelled assimilation rates using the Farquhar‐von‐Caemmerer‐Berry model (von Caemmerer, 2000) under two scenarios, where assimilation is either Rubisco‐limited *A*_*c*_ or RuBP‐regeneration‐limited *A*_*j*_:

(15) $$A_{c}=\frac{V_{c\max}\cdot \left(C_{c}-\Gamma^{*}\right)}{C_{c}+K_{M}}-R_{d},$$

(16) $$A_{j}=\frac{J\cdot \left(C_{c}-\Gamma^{*}\right)}{{4\cdot C}_{c}+8\cdot \Gamma^{*}}-R_{d},$$

where *V*_*c*max_ is the maximum Rubisco carboxylation rate, *J* is the light‐dependent whole chain electron transport rate, $K_{M}=K_{c}\cdot \left(1+O/K_{o}\right)$ is the effective Michaelis–Menten constant for Rubisco carboxylation, *K*_*c*_ and *K*_*o*_ are the Michaelis–Menten constants for [CO_2_] and [O_2_], and O is the oxygen concentration in the chloroplast (assumed to be 1.97 kPa, equal to the ambient value in the leaf chamber). Values of *K*_*c*_ and *K*_*o*_ were calculated from the leaf temperature as described previously (Bernacchi et al. 2001).

Both frameworks require values of *V*_*c*max_ and *J*, which can be estimated from the measured values of *A*_*n*_, *C*_*c*_, and *R*_*d*_ by assuming either Rubisco‐limited or RuBP‐regeneration‐limited assimilation and solving Equations (15) and (16) for *V*_*c*max_ and *J*, respectively:

(17) $$V_{\mathrm{cmax}}=\frac{\left(A_{n}+R_{d}\right)\cdot \left(C_{c}+K_{M}\right)}{C_{c}-\Gamma^{*}},$$

(18) $$J=\frac{\left(A_{n}+R_{d}\right)\cdot \left({4\cdot C}_{c}+8\cdot \Gamma^{*}\right)}{C_{c}-\Gamma^{*}}.$$

The induction curves measured here do not provide enough information to determine whether assimilation is limited by Rubisco or RuBP regeneration, so each set of limiting factors was calculated under each scenario. However, prior work with soybean cultivars have suggested Rubisco and not RubP regeneration is the predominant biochemical limitation throughout induction (Soleh et al. 2016, 2017; Taylor and Long 2017). Note, that as written, Equations (11) and (12) calculate dimensionless limiting factors, which are multiplied by 100 to express them as percentages.

### 2.6 Data Processing and Statistical Analysis

An automatic data processing and *g*_*m*_ calculation tool was developed in MATLAB (v2019a, Mathworks, https://uk.mathworks.com). MATLAB used the pretreated gas exchange data files (csv file with parameters needed for calculation) and the raw TDL data files to calculate the *g*_*m*_ through the photosynthetic induction, with the equations described earlier. The data that support the findings of this study are available at: https://doi.org/10.13012/B2IDB-7809185_V2.

**Figure 2:**
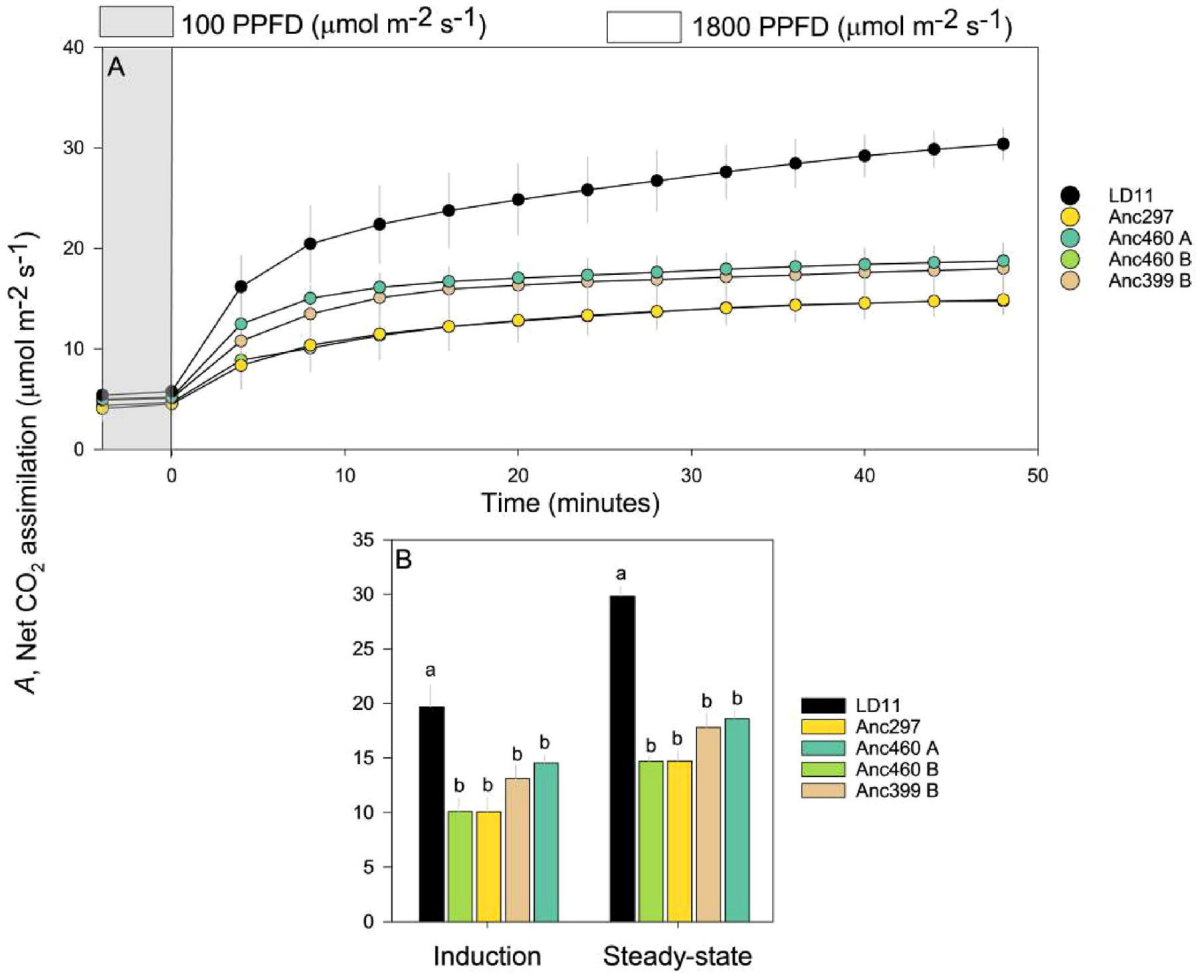
Comparisons among domesticated high‐yielding elite LD11 (*Glycine max* (L.) Merr) and four ancestor accessions (*Glycine soja* Siebold & Zucc) illustrated as (A) the average temporal response of net CO_2_ assimilation (*A*) after a transition in photosynthetic photon flux density (PPFD) from 100 (grey area) to 1800 (white area) where data points represent 4‐min averages, (B) the average response of *A* during light induction and at steady state. Light induction and steady state were defined as the initial and last 12 min (three 4‐min intervals) after an increase in light intensity, respectively. Different letters indicate significant differences (*p* < 0.1, repeated measures ANOVA, *n* = 12) and error bars indicate standard error. [Color figure can be viewed at wileyonlinelibrary.com]

**Figure 3:**
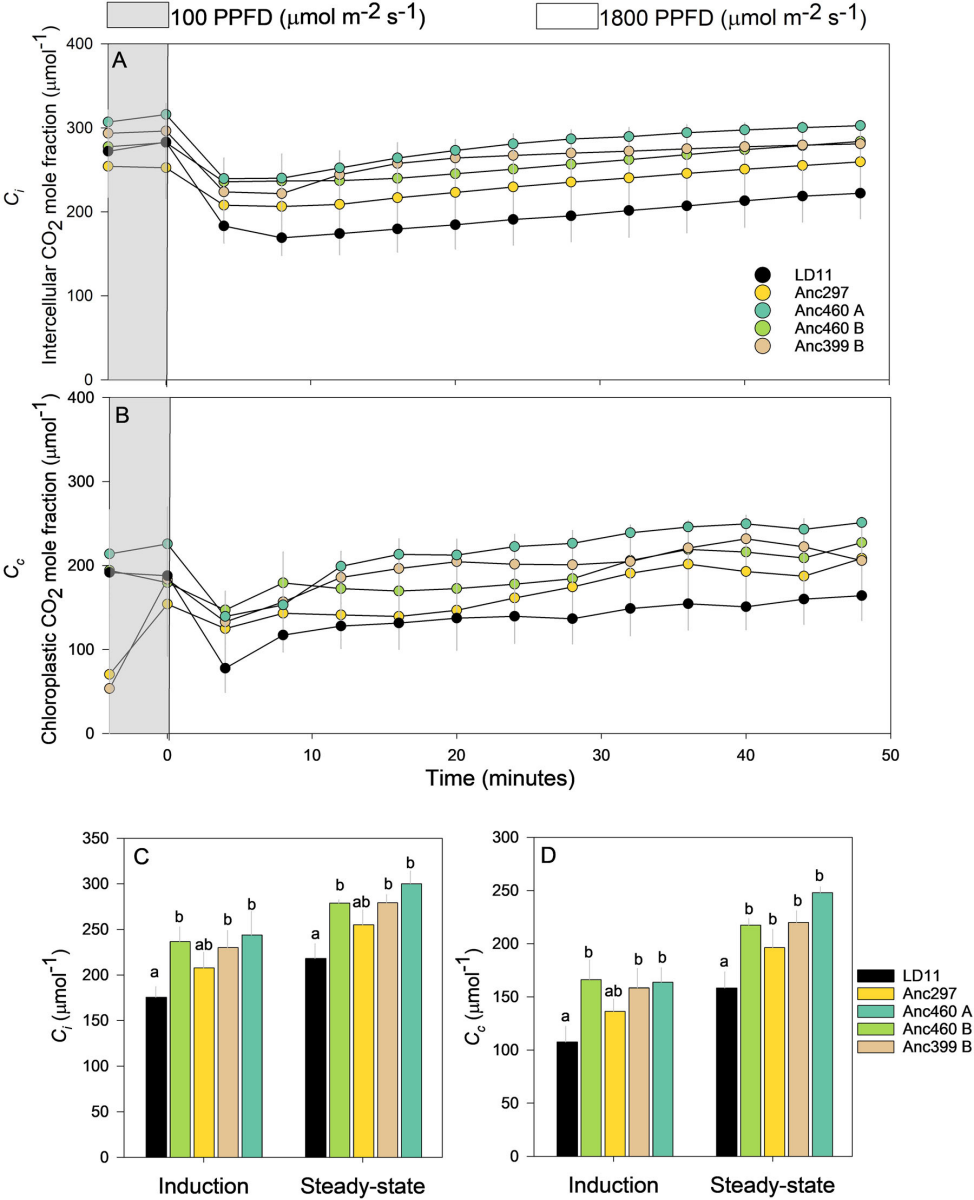
Comparisons among domesticated high‐yielding elite LD11 (*Glycine max* (L.) Merr) and four ancestor accessions (*Glycine soja* Siebold & Zucc) illustrated as the average temporal response of (A) intercellular CO_2_ mole fraction (*C*_*i*_) and (B) chloroplastic CO_2_ mole fraction (*C*_*c*_) after a transition in photosynthetic photon flux density (PPFD) from 100 (grey area) to 1800 (white area) where data points represent 4‐min averages, the average response of (C) *C*_*i*_ and (D) *C*_*c*_ during light induction and at steady state. Light induction and steady state were defined as the initial and last 12 min (three 4‐min intervals) after an increase in light intensity, respectively. Different letters indicate significant differences (*p* < 0.1, repeated measures ANOVA, *n* = 12) and error bars indicate standard error. [Color figure can be viewed at wileyonlinelibrary.com]

**Figure 4:**
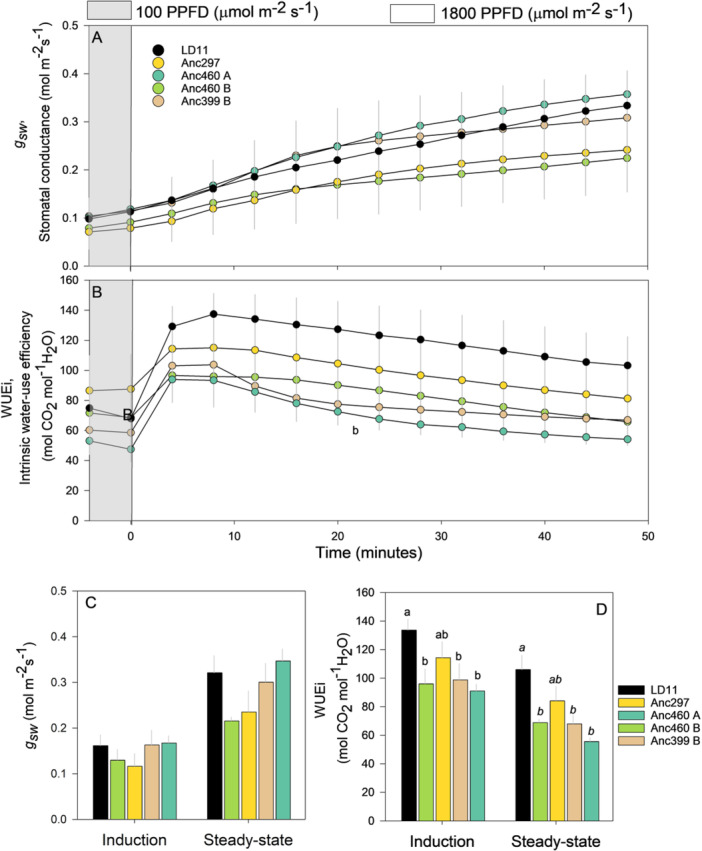
Comparisons among domesticated high‐yielding elite LD11 (*Glycine max* (L.) Merr) and four ancestor accessions (*Glycine soja* Siebold & Zucc) illustrated as the average temporal response of (A) stomatal conductance (*g*_*sw*_) and (B) intrinsic water‐use efficiency (WUEi) after a transition in photosynthetic photon flux density (PPFD) from 100 (grey area) to 1800 (white area) where data points represent 4‐min averages, the average response of (C) *g*_*sw*_ and (D) WUEi during light induction and at steady state. Light induction and steady state were defined as the initial and last 12 min (three 4‐min intervals) after an increase in light intensity, respectively. Different letters indicate significant differences (*p* < 0.1, repeated measures ANOVA, n = 12, where italic letters indicate *p* = 0.12) and error bars indicate standard error. [Color figure can be viewed at wileyonlinelibrary.com]

**Figure 5:**
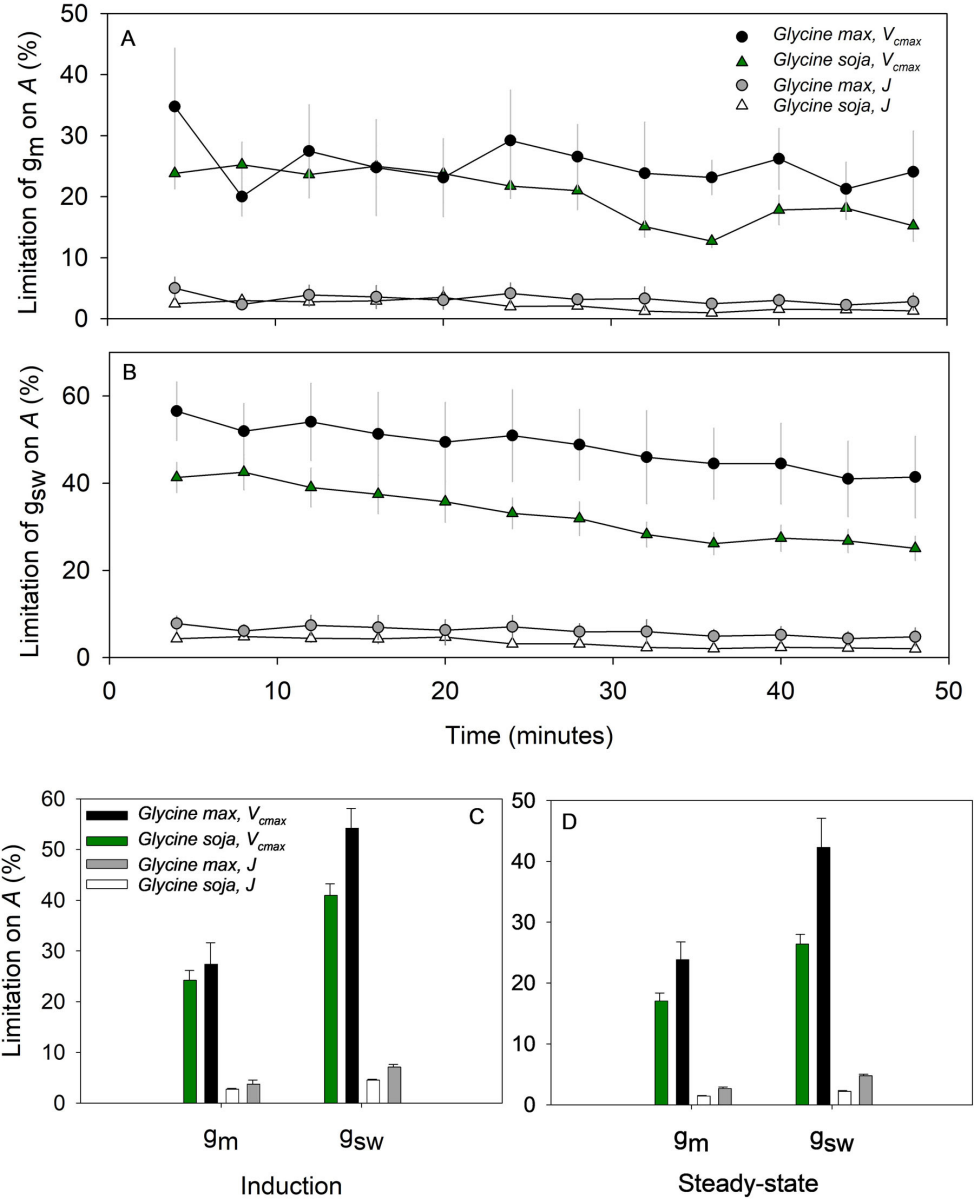
The limitation of mesophyll conductance (*g*_*m*_) and stomatal conductance (*g*_*sw*_) on net CO_2_ assimilation rate (*A*) after an increase in light intensity over time (A, B), and the average limitation during light induction (C) and steady state (D) assuming the biochemical limitations of ribulose 1,5‐biphosphosphate (RuBP) carboxylation (*V*_*c*max_) and regeneration (*J*). Limitation calculations were made using the methods described in Warren et al. (2003). The comparisons are between domesticated high‐yielding elite LD11 (*Glycine max* (L.) Merr, *n* = 4) and the average of four ancestor accessions (*Glycine soja* Siebold & Zucc, *n* = 16). Light induction and steady state were defined as the initial and last 12 min (three 4‐min intervals) after an increase in light intensity, respectively. Error bars indicate standard error. [Color figure can be viewed at wileyonlinelibrary.com]

Unreasonable values of *g*_*m*_ (*g*_*m*_ ≥ 1 or *g*_*m*_ ≤ 0 and those associated with *ξ* ≥ 50 or *ξ* ≤ 0) were removed before analysis. Data points were grouped into 4‐min intervals and the interquartile range was calculated to remove outliers within each interval before averaging. Due to noise within each replicate, the moving average of *g*_*m*_ was then calculated. Values of *A*, *C*_*i*_, *C*_*c*_, *g*_*sw*_ and WUEi were also grouped and averaged into 4‐min intervals. Only the response of photosynthetic parameters after the increase in PPFD from 100 to 1800 was evaluated; therefore, values of light induction and steady state were calculated as the average of the initial and last 12 min (three 4‐min intervals) after the increase in light intensity. Statistical analyses (*p* < 0.1 significance level) were performed using repeated measures ANOVA where accession and time were main effects. The residuals were checked for normality and constant variance, box Cox transformations were conducted if these criteria were unmet. Means comparison Dunnett test using LD11 as the control was performed if there was a significant accession effect and/or significant interaction between accession and time (R software, R Core Team 2021). Curve fitting (3 parameter Sigmoidal Hill) was conducted in SigmaPlot (SigmaPlot 15, Systat Software) for the calculation of the time taken for *g*_*m*_ to reach 50% ($t_{50g_{m}}$) and 90% ($t_{90g_{m}}$) of its steady‐state value where a one‐way ANOVA was conducted for statistical analysis (Supporting Information S1: Figures 2–6).

## 3 Results

### 3.1 The Response of Mesophyll Conductance (*g*_*m*_) and Other Photosynthetic Parameters after an Increase in Light Intensity

Steady‐state *g*_*m*_ was significantly greater (ca. 70%) in the elite modern cultivar (LD11) than the average of the four ancestors (Figure 1A,B and Supporting Information S1: Table S1). Despite attaining this higher *g*_*m*_, induction in LD11 was as rapid as in the ancestors (Figure 1C and Supporting Information S1: Figures 2‐6). Steady‐state net leaf CO_2_ assimilation rate (*A*) of LD11 was significantly higher and almost twice that of the ancestral accessions, as was the average *A* over the first 12 min of induction following transfer from low to high light (Figure 2). At steady state and throughout induction intercellular and stromal [CO_2_] (*C*_*i*_ and *C*_*c*_, respectively) were substantially lower in the modern elite (LD11) (Figure 3). *C*_*i*_ and *C*_*c*_ briefly decreased after the increase in light intensity before reaching a plateau, which was close to the shade value for the ancestral accessions, but below that for LD11 (Figure 3). In contrast to *g*_*m*_, stomatal conductance (*g*_*sw*_) was not significantly higher than the ancestral accessions, both at steady state and through induction (Figure 4). Coincided with this and the higher *A* of the elite is a higher leaf instantaneous water use efficiency (Figure 4).

### 3.2 The Limitation on Net CO_2_ Assimilation Rate (*A*)

The dynamics of the two gas diffusional limiting factors (*g*_*m*_ and g_*sw*_) affecting *A* under the assumption of RuBP regeneration (*J*) and Rubisco carboxylation (*V*_*c*max_) biochemical limitation were evaluated using the methods described in Warren et al. (2003). Since *g*_*m*_ and *A* of LD11 were found to be significantly higher than for all four ancestral accessions, the limitation comparisons were made between species where the response of all four ancestral accessions was represented by a combined average (*G. max* and *G. soja*, respectively).

Limitations analysis showed that regardless of whether RubP regeneration or Rubisco carboxylation was assumed as the biochemical limitation, the limitations on *A* imposed by both stomatal and mesophyll conductance were greater in the elite modern soybean at steady state and through induction (Figure 5). The limitations on *A* by both *g*_*m*_ and *g*_*sw*_ followed similar trends but were more severe under carboxylation than regeneration biochemical limitation through induction and at steady state (Figure 5). The limitation of *g*_*sw*_ was also greater than *g*_*m*_ under both biochemical limitation assumptions (Figure 5C,D). Limitations calculated using the methods described in Grassi and Magnani (2005), although different in scale, compare well to the conclusions from the Warren et al. (2003) method (Supporting Information S1: Figures 7AB and 8AB). The Grassi and Magnani (2005) framework also calculates biochemical limitations in addition to diffusion limitations; limitations imposed by *J* or *V*_*c*max_ on *A* were higher for *G. soja* than *G. max* during both the induction and steady‐state phases (Supporting Information S1: Figures 7C and 8C).

## 4 Discussion

This study tested two hypotheses concerning the domestication and subsequent improvement of soybean, both are strongly supported by the results obtained. (1) Mesophyll conductance (*g*_*m*_) was shown to be a significant limitation to soybean photosynthesis both at steady state and through light induction especially when the major biochemical limitation was in vivo Rubisco activity (*V*_*c*max_) (Figure 5). (2) Compared to the ancestral accessions, the elite soybean cultivar showed a near doubling and significant increase in *g*_*m*_ at both steady state and through light induction (Figure 1), which also corresponded to a substantial increase in leaf CO_2_ assimilation (*A*) and leaf level water use efficiency (WUEi, Figures 2 and 4). It also shows that domestication and breeder selection for yield had resulted in a large increase in leaf photosynthetic rate (Figure 2).

Further examination investigating the *g*_*m*_ response to light induction across a broader range of ancestral accessions as well as different domesticated accessions of soybean by year of release may strengthen the conclusions here. Previous research has explored historical soybean accessions between 1923 and 2007 where steady‐state *A*/*C*_*i*_ responses using the Variable *J* method revealed that *g*_*m*_ has not changed consistently with release date (Koester et al. 2016). Evaluation under dynamic light conditions with the isotopic discrimination technique may challenge these conclusions. We acknowledge that the equations used here to calculate *g*_*m*_ (Evans and Von Caemmerer 2013) are not the most recent. Busch et al. (2020) modified the assumptions related to the mitochondrial respiration fractionation component which improved the accuracy of *g*_*m*_ when intercellular [CO_2_] (*C*_*i*_) values are low (< 100 µmol mol^−1^). Here, *C*_*i*_ did not drop below 150 µmol mol^−1^ during light induction (Figure 3A) and Supporting Information S1: Figure 9 shows negligible differences between *g*_*m*_ calculated of Busch et al. (2020) relative to Evans and Von Caemmerer (2013) under the measurement conditions used in this study. More replicate power may also reduce the noise associated with the speed of *g*_*m*_ response to light induction (Figure 1C and Supporting Information S1: Figures 2–6). Nonetheless, such techniques require proficiency and specialized equipment where labour and costs may need to be compromised in screening large amounts of germplasm.

Mesophyll conductance is a suggested target for improvement, since it would result in higher rates of CO_2_ assimilation per unit leaf area, without any increased cost in terms of water use, so potentially providing a sustainable increase in crop productivity (Flexas et al. 2013; Lundgren and Fleming 2020; Long et al. 2022; Salesse‐Smith et al. 2024). Here, it was shown to be a substantial limitation to light‐saturated *A* at steady state (ca. 20%) and more so in the elite LD11 than the ancestral accessions when *V*_*c*,max_ was the major biochemical limitation (Figure 5). Most previous studies have estimated *g*_*m*_ under steady‐state conditions. However, the light environment in dense modern crop canopies is rarely constant, with most leaves experiencing many fluctuations in light over the course of a day (Long et al. 2022). On transition of leaves from shade to sun, *g*_*sw*_ and *g*_*m*_ increase over several minutes, and contribute to the slow increase in *A*. Under these conditions the limitation attributable to *g*_*m*_ (ca. 25%) was even greater than at steady state, showing the importance of considering light fluctuation for field crops. The limitation, at both steady state and through induction, was only slight when regeneration of RuBP, governed by the maximum rate of whole chain electron transport (*J*), is assumed the biochemical limitation (Figure 5). However, previous analyses of *A*/*C*_*i*_ responses of a range of soybean germplasm showed that in the current atmosphere, *V*_*c*,max_ and not *J*_max_ as the exclusive biochemical limitation at both light‐saturated steady‐state *A* and through light‐induction (Sakoda et al. 2016; Soleh et al. 2016, 2017). The results therefore suggest a strong limitation on assimilation by *g*_*m*_; a limitation which has decreased through selection in domestication and subsequent breeding.

As a major limitation to assimilation under current and past atmospheric [CO_2_], it might be expected that domestication and breeding would have indirectly selected for increased *g*_*m*_, particularly for the most recent soybean releases, which appear strongly source limited (Ainsworth and Long, 2021). The ca. 70% increase in *g*_*m*_ in LD11 compared to the ancestral accessions, corresponds to a near doubling of light‐saturated *A* (Figures 1 and 2) implying that *g*_*m*_ has scaled with increase in *A*. However, while increased *g*_*m*_ and the associated increased WUEi may have indirectly resulted from selection of more productive accessions, it appears that *g*_*m*_ has not kept pace with increased *A*. Despite higher *g*_*m*_ in the elite cultivar, *C*_*i*_ was ca. 60 µmol mol^−1^ lower at light‐saturated steady state (Figure 3) which would substantially increase photorespiration and decrease the rate of carboxylation. This finding implies that improvement of *g*_*m*_ has been less than the increase in biochemical capacity for CO_2_ assimilation, and suggests *g*_*m*_ is an important target for improving both productivity and water use efficiency in soybean.

Manipulation of cell wall porosity has been suggested as one means to substantially increase *g*_*m*_ (Evans 2021). In tobacco, transgenic upregulation of a pectin methyltransferase increased cell wall porosity, with concomitant significantly increased *g*_*m*_ and *A*, suggest one way to achieve this in soybean (Salesse‐Smith et al. 2024). However, the large differences in *g*_*m*_ found here between the wild ancestors and an elite line suggest that there may be substantial variation within soybean germplasm that might be exploited through marker‐assisted breeding, genomic selection, or direct selection through measurement of *g*_*m*_. Substantial variation in *g*_*m*_ and associated improvement in WUE has been shown in other crops (Barbour et al. 2010; Jahan et al. 2014; Tomás et al. 2014). In soybean, a survey of 12 cultivars showed a two‐fold variation in *g*_*m*_. This was strongly and positively correlated with variation in *A*, where 38% of the variation was due to cultivar (Tomeo and Rosenthal 2017). These results suggest *g*_*m*_ has unexplored potential within soybean breeding to deliver increased productivity and water use efficiency.

## Supporting information

Supporting information.

## Data Availability

The data that support the findings of this study are openly available in Soybean/Soja mesophyll conductance during light induction at https://doi.org/10.13012/B2IDB-7809185_V2.

## References

[pce15206-bib-0001] Acevedo‐Siaca, L. G. , R. Coe , Y. Wang , J. Kromdijk , W. P. Quick , and S. P. Long . 2020. “Variation in Photosynthetic Induction Between Rice Accessions and Its Potential for Improving Productivity.” New Phytologist 227: 1097–1108. 32124982 10.1111/nph.16454 PMC7383871

[pce15206-bib-0002] Adeniyan, O. N. , and O. T. Ayoola . 2006. “Growth and Yield Performance of Some Improved Soybean Varieties as Influenced by Intercropping with Maize and Cassava in Two Contrasting Locations in Southwest Nigeria.” African Journal of Biotechnology 5: 1886–1889.

[pce15206-bib-0003] Ainsworth, E. A. , and S. P. Long . 2021. “30 Years of Free‐Air Carbon Dioxide Enrichment (FACE): What Have We Learned about Future Crop Productivity and Its Potential for Adaptation?”Global Change Biology 27: 27–49. 33135850 10.1111/gcb.15375

[pce15206-bib-0004] Anderson, E. J. , M. L. Ali , W. D. Beavis , et al. 2019. “Soybean *Glycine max* (L.) Merr. Breeding: History, Improvement, Production and Future Opportunities.” Advances in Plant Breeding Strategies: Legumes 7: 431–516.

[pce15206-bib-0005] Barbour, M. M. , C. R. Warren , G. D. Farquhar , G. Forrester , and H. Brown . 2010. “Variability in Mesophyll Conductance Between Barley Genotypes, and Effects on Transpiration Efficiency and Carbon Isotope Discrimination.” Plant Cell Environment 33: 1176–1185. 20199618 10.1111/j.1365-3040.2010.02138.x

[pce15206-bib-0006] Bernacchi, C. J. , A. R. Portis , H. Nakano , S. von Caemmerer , and S. P. Long . 2002. “Temperature Response of Mesophyll Conductance. Implications for the Determination of Rubisco Enzyme Kinetics and for Limitations to Photosynthesis in Vivo.” Plant Physiology 130: 1992–1998. 12481082 10.1104/pp.008250 PMC166710

[pce15206-bib-0007] Bernacchi, C. J. , E. L. Singsaas , C. Pimentel , A. R. Portis, Jr. , and S. P. Long . 2001. “Improved Temperature Response Functions for Models of Rubisco‐Limited Photosynthesis.” Plant, Cell & Environment 24: 253–259.

[pce15206-bib-0008] Bowling, D. R. , S. D. Sargent , B. D. Tanner , and J. R. Ehleringer . 2003. “Tunable Diode Laser Absorption Spectroscopy for Stable Isotope Studies of Ecosystem‐Atmosphere CO­_2_ Exchange.” Agricultural and Forest Meteorology 118: 1–19.

[pce15206-bib-0009] Burgess, A. J. , C. Masclaux‐Daubresse , G. Strittmatter , et al. 2023. “Improving Crop Yield Potential: Underlying Biological Processes and Future Prospects.” Food and Energy Security 12: e435. 37035025 10.1002/fes3.435 PMC10078444

[pce15206-bib-0010] Busch, F. A. , M. Holloway‐Phillips , H. Stuart‐Williams , and G. D. Farquhar . 2020. “Revisiting Carbon Isotope Discrimination in C_3_ Plants Shows Respiration Rules When Photosynthesis Is Low.” Nature Plants 6: 245–258. 32170287 10.1038/s41477-020-0606-6

[pce15206-bib-0011] von Caemmerer, S. 2000. Biochemical Models of Leaf Photosynthesis. Clayton, VIC: CSIRO Publishing.

[pce15206-bib-0012] von Caemmerer, S. , and J. R. Evans . 2015. “Temperature Responses of Mesophyll Conductance Differ Greatly between Species.” Plant, Cell & Environment 38: 629–637. 10.1111/pce.12449 25224884

[pce15206-bib-0013] von Caemmerer, S. , and G. D. Farquhar . 1981. “Some Relationships between the Biochemistry of Photosynthesis and the Gas Exchange of Leaves.” Planta 153: 376–387. 24276943 10.1007/BF00384257

[pce15206-bib-0081] Cai, G. , and A. Brock 2021. “*The Uniform Soybean Tests: Northern Regions 2021*, 404.” West Lafyette, IN: USDA‐ARS.

[pce15206-bib-0014] Dermody, O. , S. P. Long , and E. H. DeLucia . 2006. “How Does Elevated CO_2_ or Ozone Affect the Leaf‐Area Index of Soybean When Applied Independently.” New Phytologist 169: 145–155. 16390426 10.1111/j.1469-8137.2005.01565.x

[pce15206-bib-0015] Evans, J. R. 2021. “Mesophyll Conductance: Walls, Membranes and Spatial Complexity.” New Phytologist 229: 1864–1876. 33135193 10.1111/nph.16968

[pce15206-bib-0016] Evans, J. R. , and S. Von Caemmerer . 2013. “Temperature Response of Carbon Isotope Discrimination and Mesophyll Conductance in Tobacco.” Plant, Cell & Environment 36: 745–756. 10.1111/j.1365-3040.2012.02591.x 22882584

[pce15206-bib-0017] Evans, J. R. , T. D. Sharkey , J. A. Berry , and G. D. Farquhar . 1986. “Carbon Isotope Discrimination Measured Concurrently with Gas‐Exchange to Investigate CO_2_ Diffusion in Leaves of Higher Plants.” Australian Journal of Plant Physiology 13: 281–292.

[pce15206-bib-0018] Farquhar, G. D. , and L. A. Cernusak . 2012. “Ternary Effects on the Exchange of Isotopologues of Carbon Dioxide.” Plant, Cell & Environment 35: 1221–1231. 10.1111/j.1365-3040.2012.02484.x 22292425

[pce15206-bib-0019] Flexas, J. , M. Carriquí , and F. J. Cano , et al. 2018. “CO2 Diffusion Inside Photosynthetic Organs.” In Leaf: A Platform for Performing Photosynthesis, edited by W. W. AdamsI and I. Terashima , 163–208. ‎ Springer International Publishing AG.

[pce15206-bib-0020] Flexas, J. , Ü. Niinemets , A. Gallé , et al. 2013. “Diffusional Conductances to CO_2_ as a Target for Increasing Photosynthesis and Photosynthetic Water‐Use Efficiency.” Photosynthesis Research 117: 45–59. 23670217 10.1007/s11120-013-9844-z

[pce15206-bib-0021] Flexas, J. , M. Ribas‐carbó , A. Diaz‐espejo , J. Galmés , and H. Medrano . 2008. “Mesophyll Conductance to CO_2_: Current Knowledge and Future Prospects.” Plant, Cell & Environment 31: 602–621. 10.1111/j.1365-3040.2007.01757.x 17996013

[pce15206-bib-0022] Grassi, G. , and F. Magnani . 2005. “Stomatal, Mesophyll Conductance and Biochemical Limitations to Photosynthesis as Affected by Drought and Leaf Ontogeny in Ash and Oak Trees.” Plant, Cell & Environment 28: 834–849.

[pce15206-bib-0023] Harley, P. C. , F. Loreto , G. Dimarco , and T. D. Sharkey . 1992. “Theoretical Considerations When Estimating the Mesophyll Conductance to CO_2_ Flux by Analysis of the Response of Photosynthesis to CO_2_ .” Plant Physiology 98: 1429–1436. 16668811 10.1104/pp.98.4.1429 PMC1080368

[pce15206-bib-0024] Jahan, E. , J. S. Amthor , G. D. Farquhar , R. Trethowan , and M. M. Barbour . 2014. “Variation in Mesophyll Conductance Among Australian Wheat Genotypes.” Functional Plant Biology 41: 568–580. 32481014 10.1071/FP13254

[pce15206-bib-0025] Jaikumar, N. S. , S. S. Stutz , S. B. Fernandes , et al. 2021. “Can Improved Canopy Light Transmission Ameliorate Loss of Photosynthetic Efficiency in the Shade? An Investigation of Natural Variation in *Sorghum bicolor* .” Journal of Experimental Botany 72: 4965–4980. 33914063 10.1093/jxb/erab176 PMC8219039

[pce15206-bib-0026] Kaiser, E. , J. Kromdijk , J. Harbinson , E. Heuvelink , and L. F. M. Marcelis . 2017. “Photosynthetic Induction and Its Diffusional, Carboxylation and Electron Transport Processes as Affected by CO_2_ Partial Pressure, Temperature, Air Humidity and Blue Irradiance.” Annals of Botany 119: 191–205. 28025286 10.1093/aob/mcw226 PMC5218377

[pce15206-bib-0027] Kamara, A. Y. , A. I. Tofa , T. Ademulegun , et al. 2019. “Maize‐Soybean Intercropping for Sustainable Intensification of Cereal‐Legume Cropping Systems in Northern Nigeria.” Experimental Agriculture 55: 73–87.

[pce15206-bib-0028] Kim, M. Y. , K. Van , Y. J. Kang , K. H. Kim , and S.‐H. Lee . 2012. “Tracing Soybean Domestication History: From Nucleotide to Genome.” Breeding Science 61: 445–452. 23136484 10.1270/jsbbs.61.445 PMC3406779

[pce15206-bib-0029] Koester, R. P. , B. M. Nohl , B. W. Diers , and E. A. Ainsworth . 2016. “Has Photosynthetic Capacity Increased with 80 years of Soybean Breeding? An Examination of Historical Soybean Cultivars.” Plant, Cell & Environment 39: 1058–1067. 10.1111/pce.12675 26565891

[pce15206-bib-0030] Lanigan, G. J. , N. Betson , H. Griffiths , and U. Seibt . 2008. “Carbon Isotope Fractionation during Photorespiration and Carboxylation in *Senecio* .” Plant Physiology 148: 2013–2020. 18923019 10.1104/pp.108.130153 PMC2593675

[pce15206-bib-0031] Leverett, A. , and J. Kromdijk . 2024. “The Long and Tortuous Path Towards Improving Photosynthesis by Engineering Elevated Mesophyll Conductance.” Plant, Cell & Environment 47: 3411–3427. 10.1111/pce.14940 38804598

[pce15206-bib-0032] Li, C. , E. Hoffland , T. W. Kuyper , et al. 2020. “Syndromes of Production in Intercropping Impact Yield Gains.” Nature Plants 6: 653–660. 32483328 10.1038/s41477-020-0680-9

[pce15206-bib-0033] Liu, T. , M. M. Barbour , D. Yu , S. Rao , and X. Song . 2022. “Mesophyll Conductance Exerts a Significant Limitation on Photosynthesis during Light Induction.” New Phytologist 233: 360–372. 34601732 10.1111/nph.17757

[pce15206-bib-0034] Liu, X. , J. He , Y. Wang , et al. 2020. “Geographic Differentiation and Phylogeographic Relationships Among World Soybean Populations.” Crop Journal 8: 260–272.

[pce15206-bib-0035] Lochocki, E. B. 2024. “PhotoGEA: Photosynthetic Gas Exchange Analysis.” R package version 1.0.0”. https://eloch216.github.io/PhotoGEA.

[pce15206-bib-0036] Long, S. P. , A. Marshall‐Colon , and X. G. Zhu . 2015. “Meeting the Global Food Demand of the Future by Engineering Crop Photosynthesis and Yield Potential.” Cell 161: 56–66. 25815985 10.1016/j.cell.2015.03.019

[pce15206-bib-0037] Long, S. P. , S. H. Taylor , S. J. Burgess , et al. 2022. “Into the Shadows and Back into Sunlight: Photosynthesis in Fluctuating Light.” Annual Review of Plant Biology 73: 617–648. 10.1146/annurev-arplant-070221-024745 35595290

[pce15206-bib-0038] Lundgren, M. R. , and A. J. Fleming . 2020. “Cellular Perspectives for Improving Mesophyll Conductance.” The Plant Journal 101: 845–857. 31854030 10.1111/tpj.14656 PMC7065256

[pce15206-bib-0039] Mbah, E. U. , C. O. Muoneke , and D. A. Okpara . 2009. “Evaluation of Cassava *Manihot esculenta Manihot Esculenta* (Crantz) Planting Methods and Soybean *Glycine max* (L.) Merrill Sowing Dates on the Yield Performance of the Component Species in Cassava/Soybean Intercrop under the Humid Tropical Lowlands of Southeastern Nigeria.” African Journal of Biotechnology 8: 42–47.

[pce15206-bib-0040] McAusland, L. , S. Vialet‐Chabrand , P. Davey , N. R. Baker , O. Brendel , and T. Lawson . 2016. “Effects of Kinetics of Light‐Induced Stomatal Responses on Photosynthesis and Water‐Use Efficiency.” New Phytologist 211: 1209–1220. 27214387 10.1111/nph.14000 PMC4982059

[pce15206-bib-0041] Murchie, E. H. , M. Pinto , and P. Horton . 2009. “Agriculture and the New Challenges for Photosynthesis Research.” New Phytologist 181: 532–552. 19140947 10.1111/j.1469-8137.2008.02705.x

[pce15206-bib-0042] Pearcy, R. W. 1990. “Sunflecks and Photosynthesis in Plant Canopies.” Annual Review of Plant Physiology and Plant Molecular Biology 41: 421–453.

[pce15206-bib-0043] Pelech, E. A. , B. C. S. Alexander , and C. J. Bernacchi . 2021. “Photosynthesis, Yield, Energy Balance, and Water‐Use of Intercropped Maize and Soybean.” Plant Direct 5, no. 12: e365. 34938940 10.1002/pld3.365 PMC8671796

[pce15206-bib-0044] Pelech, E. A. , J. B. Evers , T. L. Pederson , D. W. Drag , P. Fu , and C. J. Bernacchi . 2023. “Leaf, Plant, to Canopy: A Mechanistic Study on Aboveground Plasticity and Plant Density Within a Maize‐Soybean Intercrop System for the Midwest, USA.” Plant, Cell & Environment 46: 405–421. 10.1111/pce.14487 PMC10100491 36358006

[pce15206-bib-0082] R Core Team . 2021. R: A Language and Environment for Statistical Computing. Vienna, Austria: R Foundation for Statistical Computing. http://www.R‐project.org/.

[pce15206-bib-0045] Roeske, C. A. , and M. H. O'Leary . 1984. “Carbon Isotope Effects on Enzyme‐Catalyzed Carboxylation of Ribulose Bisphosphate.” Biochemistry 23: 6275–6284. 10.1021/bi00328a005 3924094

[pce15206-bib-0046] Sakoda, K. , Y. Tanaka , S. P. Long , and T. Shiraiwa . 2016. “Genetic and Physiological Diversity in the Leaf Photosynthetic Capacity of Soybean.” Crop Science 56: 2731–2741.

[pce15206-bib-0047] Sakoda, K. , W. Yamori , M. Groszmann , and J. R. Evans . 2021. “Stomatal, Mesophyll Conductance, and Biochemical Limitations to Photosynthesis during Induction.” Plant Physiology 185: 146–160. 33631811 10.1093/plphys/kiaa011 PMC8133641

[pce15206-bib-0048] Salesse‐Smith, C. E. , S. M. Driever , and V. C. Clarke . 2022. “Modifying Mesophyll Conductance to Optimise Photosynthesis in Crops.” In Burleigh Dodds Series In Agricultural Science, Vol. 27. Sawston, UK: Burleigh Dodds Science Publishing.

[pce15206-bib-0049] Salesse‐Smith, C. E. , E. B. Lochocki , L. Doran , B. E. Haas , S. S. Stutz , and S. P. Long . 2024. “Greater Mesophyll Conductance and Leaf Photosynthesis in the Field through Modified Cell Wall Porosity and Thickness via AtCGR3 Expression in Tobacco.” Plant Biotechnology Journal 22: 2504–2517. 38687118 10.1111/pbi.14364 PMC11331791

[pce15206-bib-0050] Soleh, M. A. , Y. Tanaka , S. Y. Kim , S. C. Huber , K. Sakoda , and T. Shiraiwa . 2017. “Identification of Large Variation in the Photosynthetic Induction Response Among 37 Soybean [*Glycine max* (L.) Merr.] Genotypes That Is not Correlated with Steady‐State Photosynthetic Capacity.” Photosynthesis Research 131: 305–315. 27878416 10.1007/s11120-016-0323-1

[pce15206-bib-0051] Soleh, M. A. , Y. Tanaka , Y. Nomoto , et al. 2016. “Factors Underlying Genotypic Differences in the Induction of Photosynthesis in Soybean [*Glycine max* (L.) Merr.].” Plant, Cell & Environment 39: 685–693. 10.1111/pce.12674 26538465

[pce15206-bib-0052] De Souza, A. P. , Y. Wang , D. J. Orr , E. Carmo‐Silva , and S. P. Long . 2020. “Photosynthesis Across African Cassava Germplasm Is Limited by Rubisco and Mesophyll Conductance at Steady State, but by Stomatal Conductance in Fluctuating Light.” New Phytologist 225: 2498–2512. 31446639 10.1111/nph.16142 PMC7065220

[pce15206-bib-0053] Specht, J. E. , B. W. Diers , R. L. Nelson , J. F. Ferraz de Toledo , J. A. Torrion , and P. Grassini . 2014. “Soybean.” In Yield Gains in Major U.S. Field Crops, edited by S. Smith , B. Diers , J. Specht , and B. Carver , 311–355.

[pce15206-bib-0054] Taylor, S. H. , and S. P. Long . 2017. “Slow Induction of Photosynthesis on Shade to Sun Transitions in Wheat May Cost at Least 21% of Productivity.” Philosophical Transactions of the Royal Society B: Biological Sciences 372: 20160543. 10.1098/rstb.2016.0543 PMC5566890 28808109

[pce15206-bib-0055] Tazoe, Y. , S. VON Caemmerer , G. M. Estavillo , and J. R. Evans . 2011. “Using Tunable Diode Laser Spectroscopy to Measure Carbon Isotope Discrimination and Mesophyll Conductance to CO_2_ Diffusion Dynamically at Different CO_2_ Concentrations.” Plant, Cell & Environment 34: 580–591. 10.1111/j.1365-3040.2010.02264.x 21251018

[pce15206-bib-0056] Tomás, M. , H. Medrano , E. Brugnoli , et al. 2014. “Variability of Mesophyll Conductance in Grapevine Cultivars under Water Stress Conditions in Relation to Leaf Anatomy and Water Use Efficiency.” Australian Journal of Grape and Wine Research 20: 272–280.

[pce15206-bib-0057] Tomeo, N. J. , and D. M. Rosenthal . 2017. “Variable Mesophyll Conductance Among Soybean Cultivars Sets a Tradeoff Between Photosynthesis and Water‐Use‐Efficiency.” Plant Physiology 174: 241–257. 28270627 10.1104/pp.16.01940 PMC5411144

[pce15206-bib-0058] Ubierna, N. , W. Sun , D. M. Kramer , and A. B. Cousins . 2013. “The Efficiency of C_4_ Photosynthesis under Low Light Conditions in *Zea mays*, *Miscanthus* x *giganteus* and *Flaveria bidentis* .” Plant, Cell & Environment 36: 365–381. 10.1111/j.1365-3040.2012.02579.x 22812384

[pce15206-bib-0059] Wang, Y. , S. J. Burgess , E. M. de Becker , and S. P. Long . 2020. “Photosynthesis in the Fleeting Shadows: an Overlooked Opportunity for Increasing Crop Productivity.” Plant Journal 101: 874–884. 10.1111/tpj.14663 PMC7064922 31908116

[pce15206-bib-0060] Wang, Y. , S. S. Stutz , C. J. Bernacchi , R. A. Boyd , D. R. Ort , and S. P. Long . 2022. “Increased Bundle‐Sheath Leakiness of CO_2_ during Photosynthetic Induction Shows a Lack of Coordination between the C_4_ and C_3_ Cycles.” New Phytologist 236: 1661–1675. 36098668 10.1111/nph.18485 PMC9827928

[pce15206-bib-0061] Warren, C. R. , G. J. Ethier , N. J. Livingston , et al. 2003. “Transfer Conductance in Second Growth Douglas‐Fir (*Pseudotsuga menziesii* (Mirb.)Franco) Canopies.” Plant, Cell & Environment 26: 1215–1227.

[pce15206-bib-0062] Williams, D. R. , M. Clark , G. M. Buchanan , G. F. Ficetola , C. Rondinini , and D. Tilman . 2021. “Proactive Conservation to Prevent Habitat Losses to Agricultural Expansion.” Nature Sustainability 4: 314–322.

