# Supplementary material for: Have We Selected for Higher Mesophyll Conductance in Domesticating Soybean?
Source: Plant Cell Environ. 2024 Oct 27;48(2):1594–607. doi: 10.1111/pce.15206 (PMC11695774; doi:10.1111/pce.15206)
Supplement: Supplementary file 1 — Supporting information. [file PCE-48-1594-s001.docx]

| **Supplementary Table 1 \| Repeated measures ANOVA and Dunnett means comparison results (α = 0.1) for gas exchange parameters between domesticated high-yielding elite LD11 (*Glycine max* (L.) Merr) and four ancestor accessions (*Glycine soja* Siebold & Zucc).** | | | |
| --- | --- | --- | --- |
| **Parameter** | **Factor** | **Repeated measures ANOVA** | **Dunnett test** |
| g_m_ induction | Accession | <0.1 | LD11 - Anc297, Anc399B, Anc460A, Anc460B |
|  | Time | <0.01 |  |
|  | Interaction | <0.05 |  |
| g_m_ steady state | Accession | <0.01 | LD11 - Anc297, Anc399B, Anc460A, Anc460B |
|  | Time | <0.01 |  |
|  | Interaction | ns |  |
| *A* induction | Accession | <0.05 | LD11 - Anc297, Anc399B, Anc460A, Anc460B |
|  | Time | <0.01 |  |
|  | Interaction | <0.01 |  |
| *A* steady state | Accession | <0.01 | LD11 - Anc297, Anc399B, Anc460A, Anc460B |
|  | Time | <0.01 |  |
|  | Interaction | ns |  |
| Ci induction | Accession | <0.05 | LD11 - Anc399B, Anc460A, Anc460B |
|  | Time | ns |  |
|  | Interaction | ns |  |
| Ci steady state | Accession | = 0.1 | LD11 - Anc297, Anc399B, Anc460A, Anc460B |
|  | Time | <0.01 |  |
|  | Interaction | ns |  |
| g_sw_ induction | Accession | ns | - |
|  | Time | <0.01 |  |
|  | Interaction | ns |  |
| g_sw_ steady state | Accession | ns | - |
|  | Time | <0.1 |  |
|  | Interaction | ns |  |
| Cc induction | Accession | <0.1 | LD11 - Anc399B, Anc460A, Anc460B |
|  | Time | <0.01 |  |
|  | Interaction | ns |  |
| Cc steady-state | Accession | <0.1 | LD11 - Anc297, Anc399B, Anc460A, Anc460B |
|  | Time | ns |  |
|  | Interaction | <0.01 |  |
| WUEi induction | Accession | <0.05 | LD11 - Anc399B, Anc460A, Anc460B |
|  | Time | ns |  |
|  | Interaction | ns |  |
| WUEi steady state | Accession | 0.12 | LD11 - Anc399B, Anc460A, Anc460B |
|  | Time | <0.01 |  |
|  | Interaction | ns |  |
| *Results are reported for mesophyll conductance (g_m_), net CO_2_ assimilation rate (A), intercellular and chloroplastic CO_2_ mole fraction (C_i_ and C_c_), stomatal conductance (g_sw_), and intrinsic water-use efficiency (WUEi). Values of induction and steady state are the average of the initial and last 12 minutes (three 4-minute intervals) after an increase in light intensity, respectively.* | | | |

Supplementary Figure 1 – Photograph of (A) clear-top leaf chamber enclosed on a single leaf from a *Glycine soja* Siebold & Zucc accession with a 6 cm^2^ leaf area, and (B) closed clear-top leaf chamber with small light source attached enclosed on a *Glycine soja* Siebold & Zucc accession.

**B**


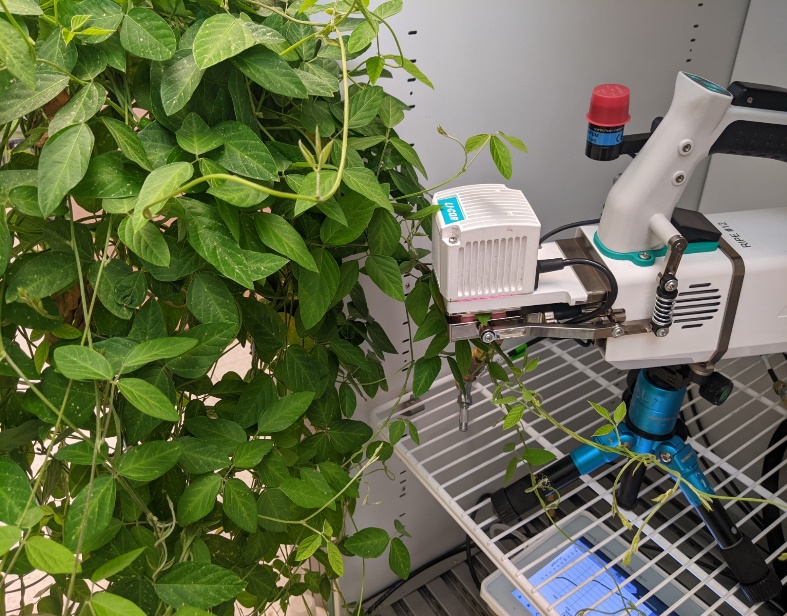


**A**


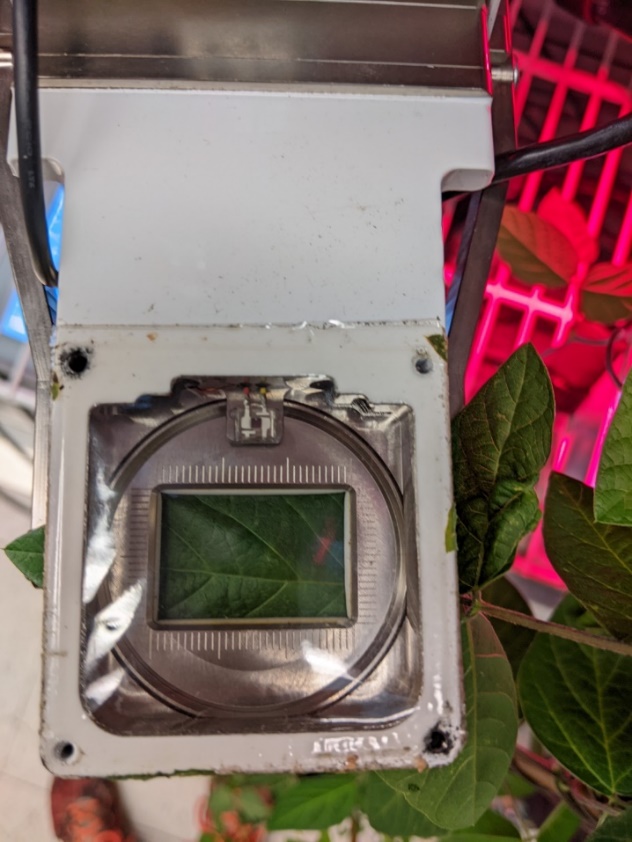


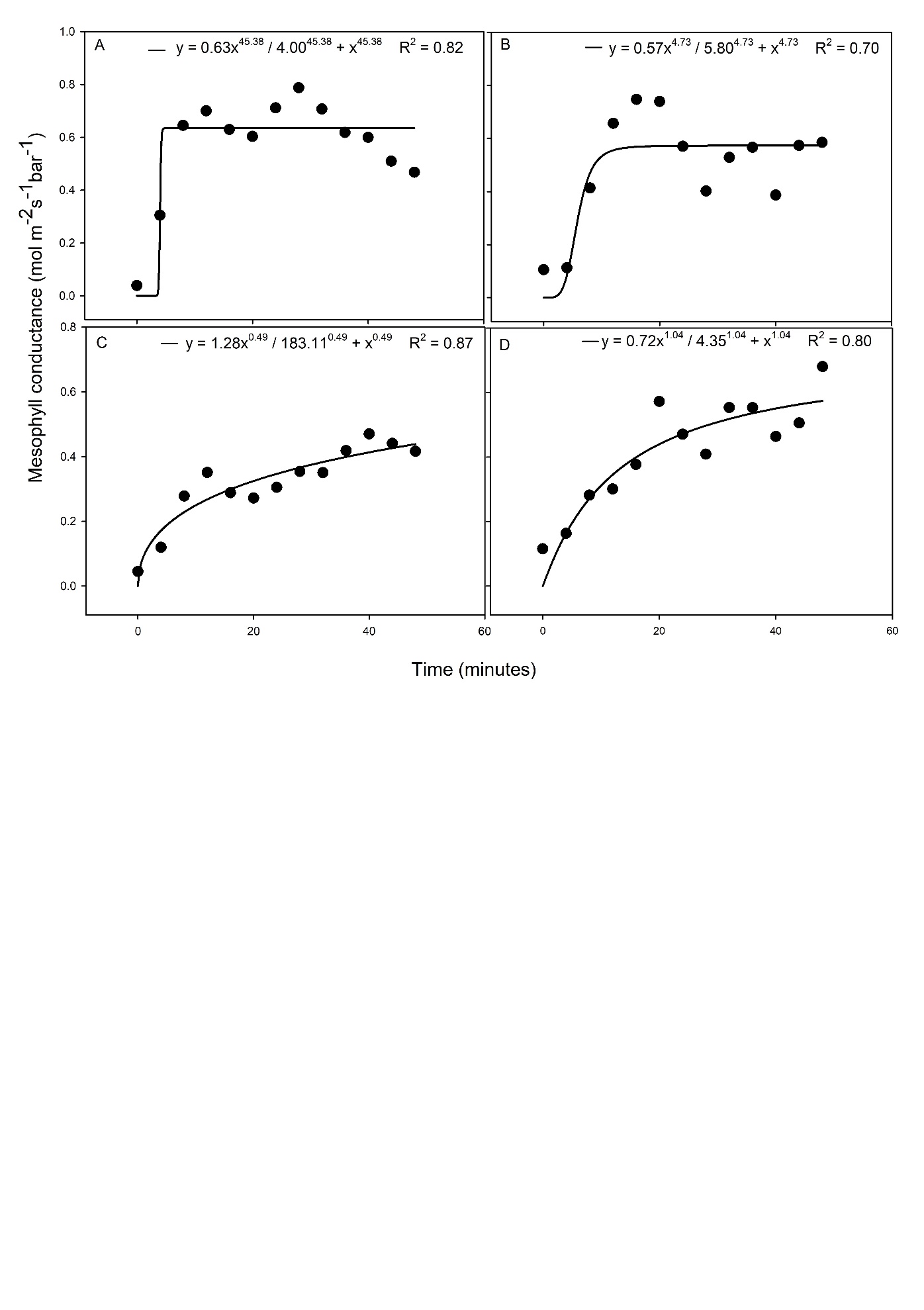
Supplementary Figure 2 – Curve fitting regressions of mesophyll conductance over time after an increase in photosynthetic photon flux density (PPFD) to calculate the time taken to reach 50% (t_50gm_) and 90% (t_90gm_) of its steady-state value for each replicate (A-D) of domesticated high-yielding elite LD11 (*Glycine max* (L.) Merr). Data points indicate the moving average and solid black lines represent the sigmoidal Hill regression between mesophyll conductance and time. The Hill equation and R^2^ for each regression is indicated within each graph.


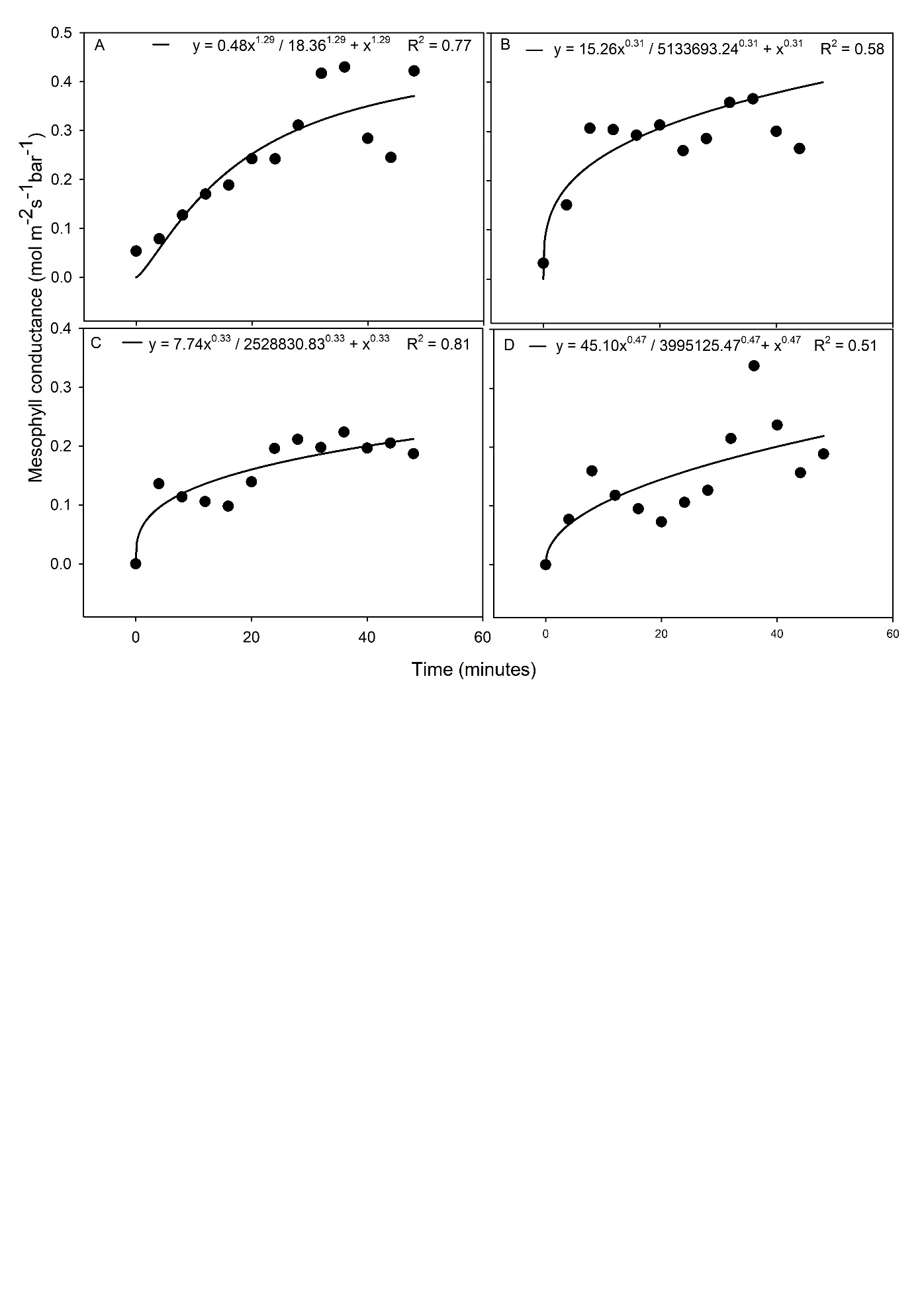
Supplementary Figure 3 – Curve fitting regressions of mesophyll conductance over time after an increase in photosynthetic photon flux density (PPFD) to calculate the time taken to reach 50% (t_50gm_) and 90% (t_90gm_) of its steady-state value for each replicate (A-D) of ancestor accession Anc297 (*Glycine soja* Siebold & Zucc). Data points indicate the moving average and solid black lines represent the sigmoidal Hill regression between mesophyll conductance and time. The Hill equation and R^2^ for each regression is indicated within each graph.


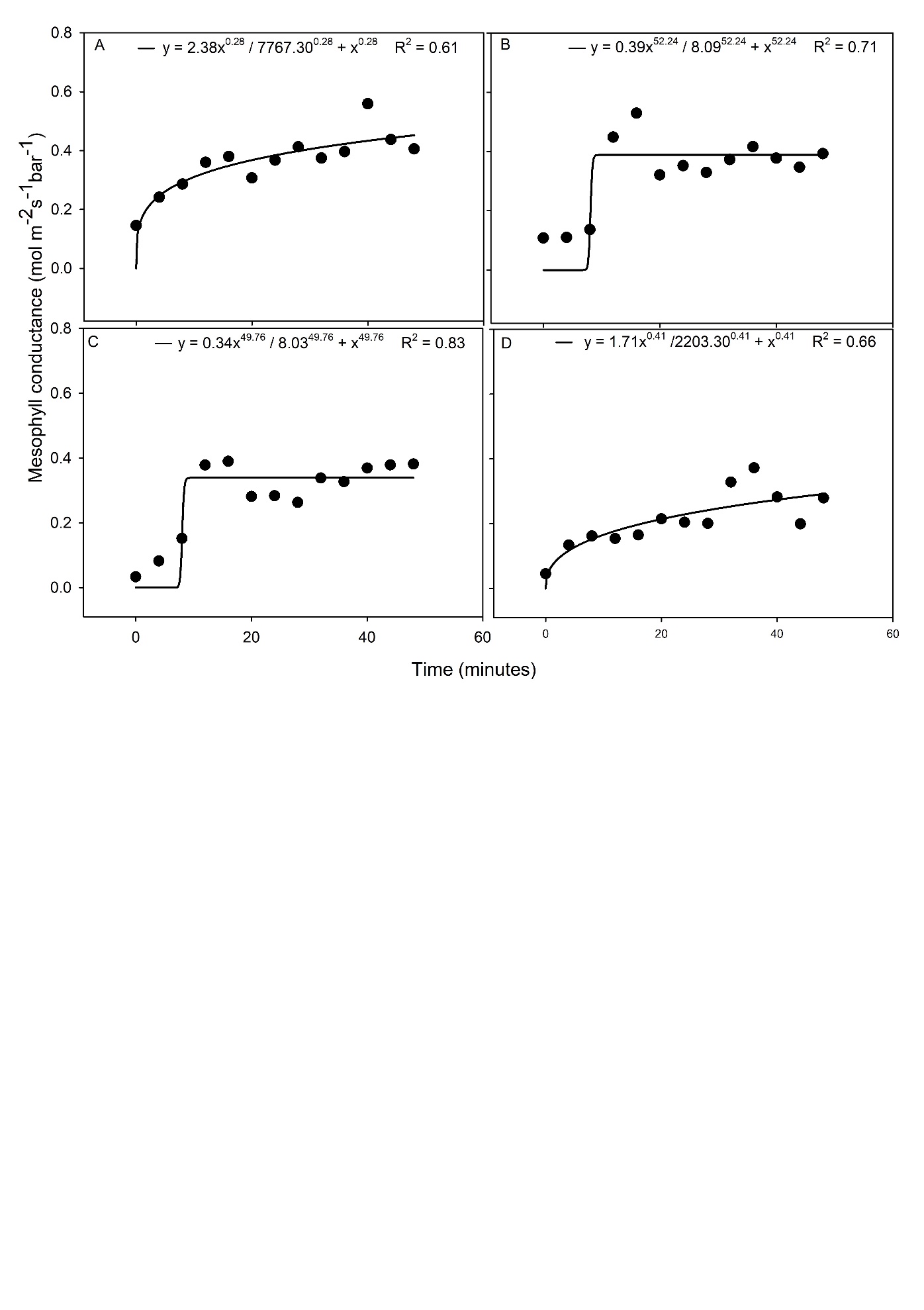
Supplementary Figure 4 – Curve fitting regressions of mesophyll conductance over time after an increase in photosynthetic photon flux density (PPFD) to calculate the time taken to reach 50% (t_50gm_) and 90% (t_90gm_) of its steady-state value for each replicate (A-D) of ancestor accession Anc460 A (*Glycine soja* Siebold & Zucc). Data points indicate the moving average and solid black lines represent the sigmoidal Hill regression between mesophyll conductance and time. The Hill equation and R^2^ for each regression is indicated within each graph.


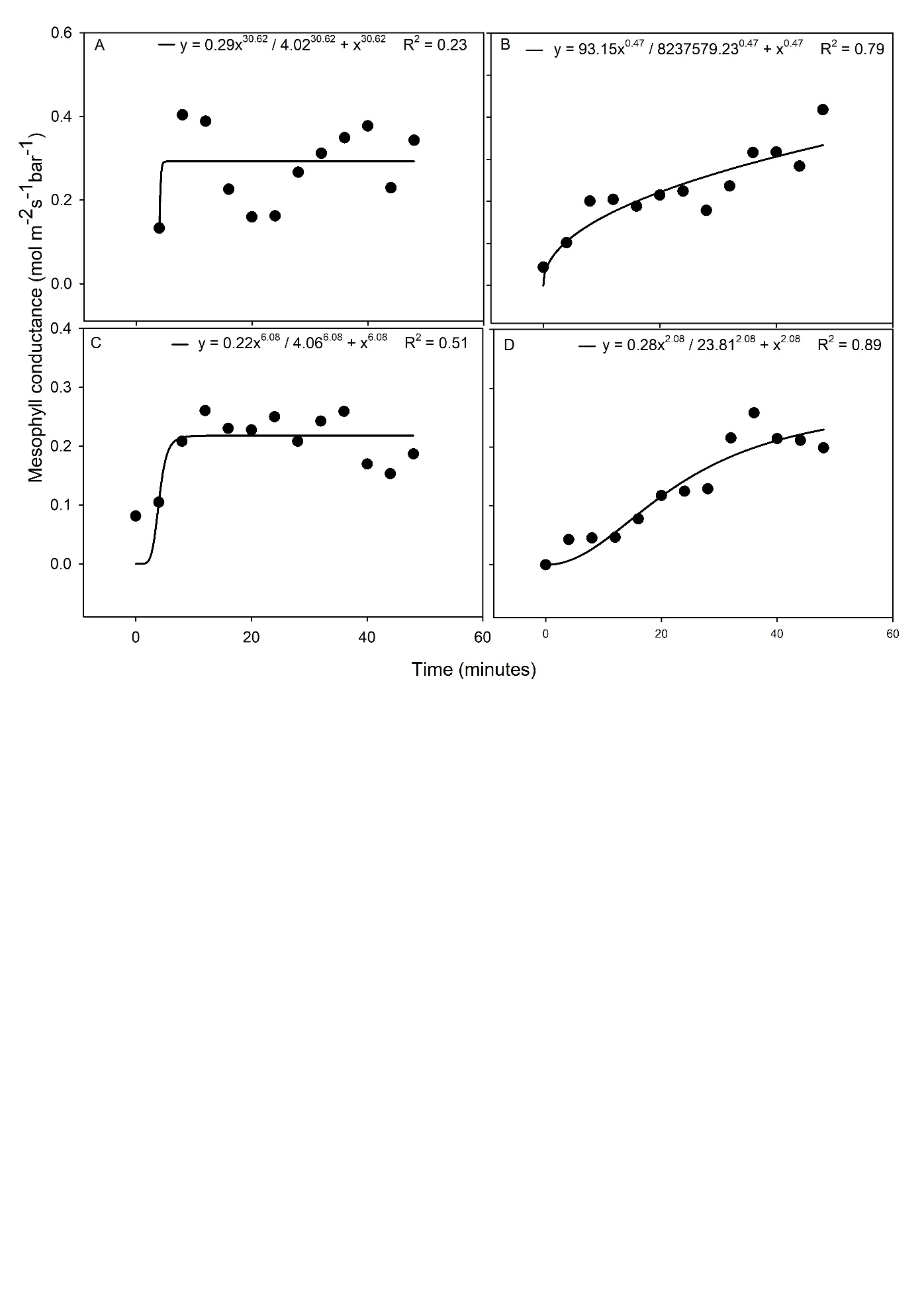
Supplementary Figure 5 – Curve fitting regressions of mesophyll conductance over time after an increase in photosynthetic photon flux density (PPFD) to calculate the time taken to reach 50% (t_50gm_) and 90% (t_90gm_) of its steady-state value for each replicate (A-D) of ancestor accession Anc460 B (*Glycine soja* Siebold & Zucc). Data points indicate the moving average and solid black lines represent the sigmoidal Hill regression between mesophyll conductance and time. The Hill equation and R^2^ for each regression is indicated within each graph.


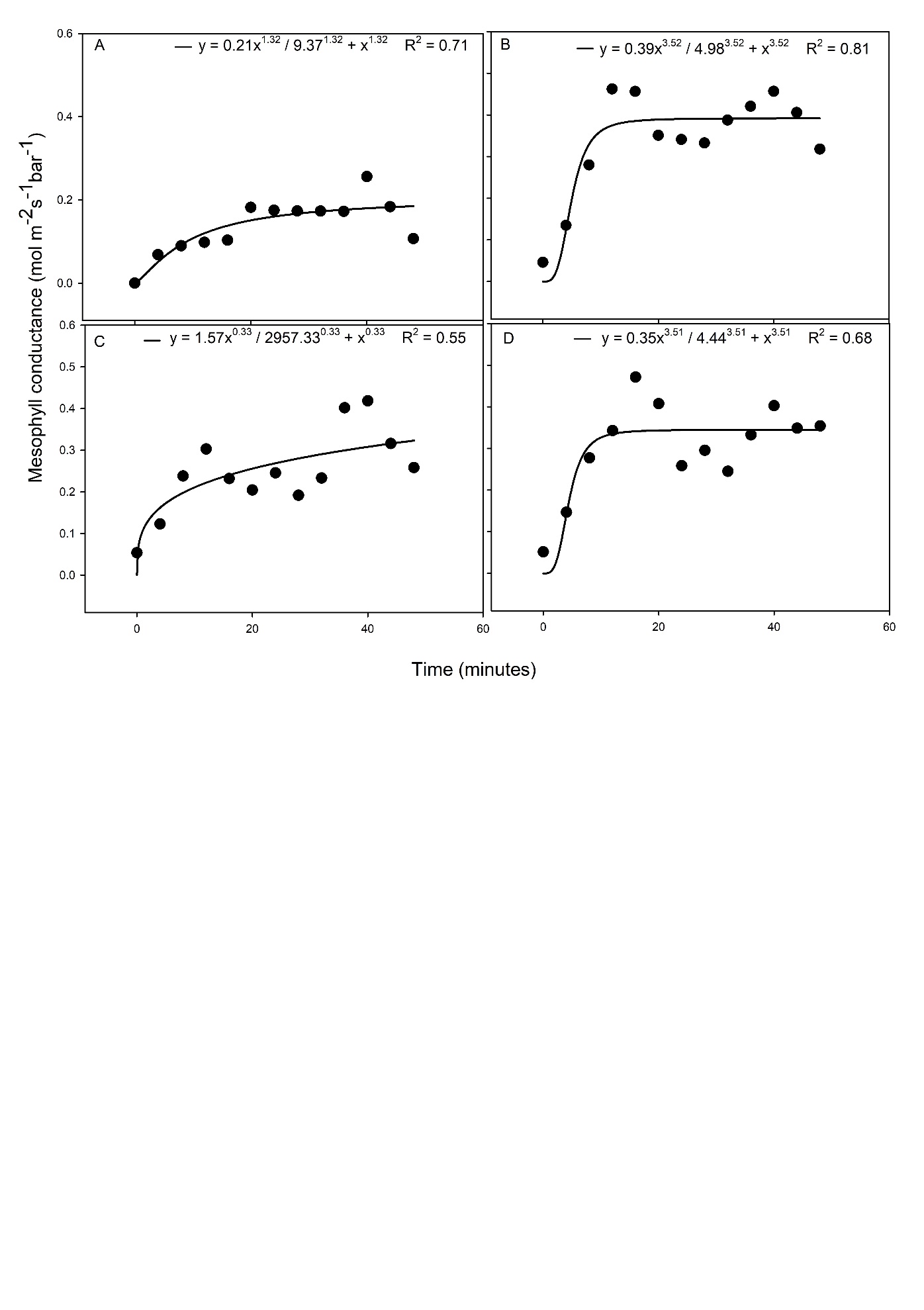
Supplementary Figure 6 – Curve fitting regressions of mesophyll conductance over time after an increase in photosynthetic photon flux density (PPFD) to calculate the time taken to reach 50% (t_50gm_) and 90% (t_90gm_) of its steady-state value for each replicate (A-D) of ancestor accession Anc399 B (*Glycine soja* Siebold & Zucc). Data points indicate the moving average and solid black lines represent the sigmoidal Hill regression between mesophyll conductance and time. The Hill equation and R^2^ for each regression is indicated within each graph.


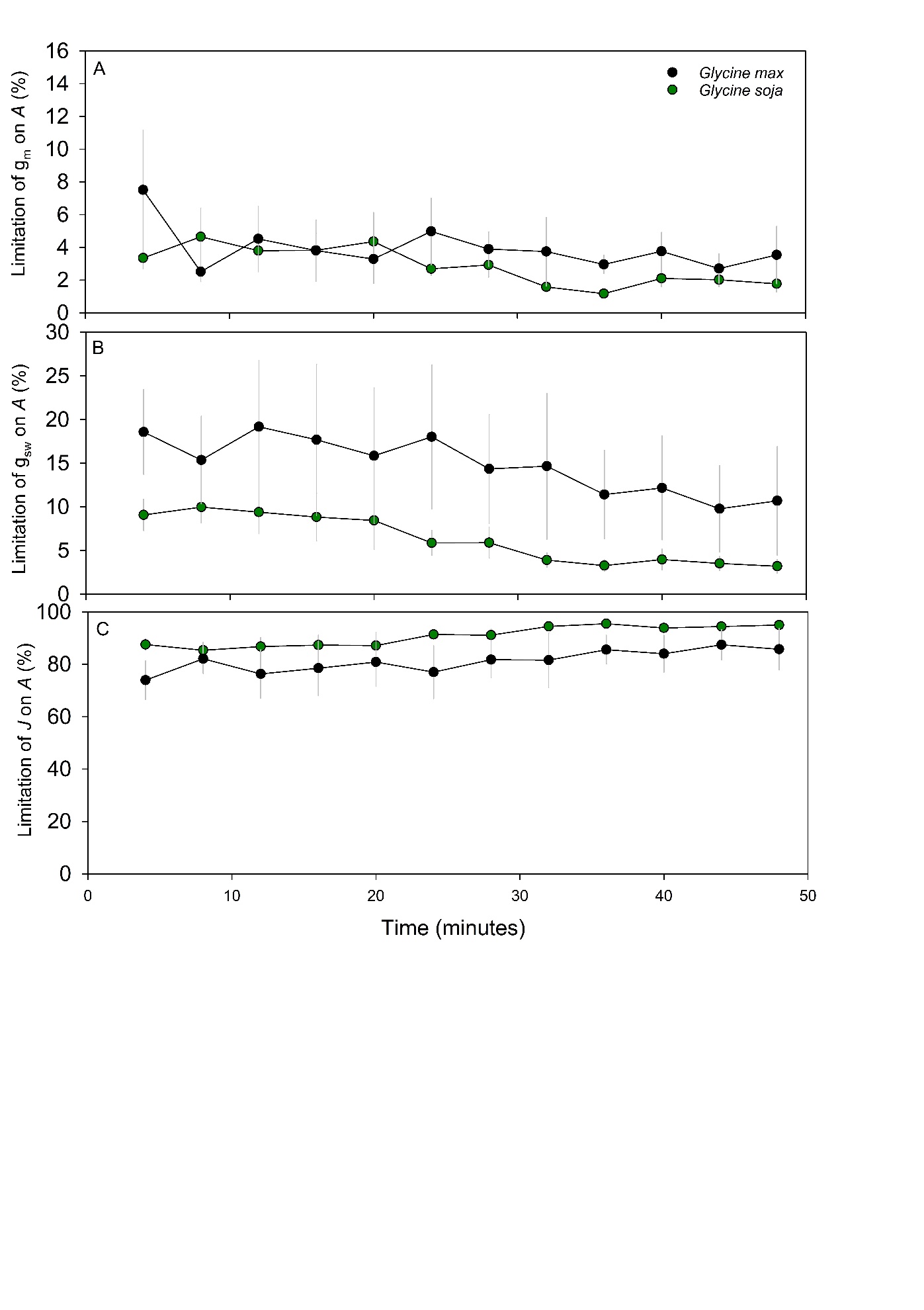
Supplementary Figure 7 – The limitation of mesophyll conductance (g_m_, A), stomatal conductance (g_sw,_, B) and electron transport rate for the regeneration of Ribulose 1,5-biphosphate ([RuBP] *J*, C) on net CO_2_ assimilation rate (*A*) after an increase in light intensity over time assuming the biochemical limitation of RuBP regeneration**.** Limitation calculations were made using the methods described in Grassi & Magnani, 2005. The comparisons are between domesticated high-yielding elite LD11 (*Glycine max* (L.) Merr, n = 4) and the average of four ancestor accessions (*Glycine soja* Siebold & Zucc, n = 16). Error bars indicate standard error.
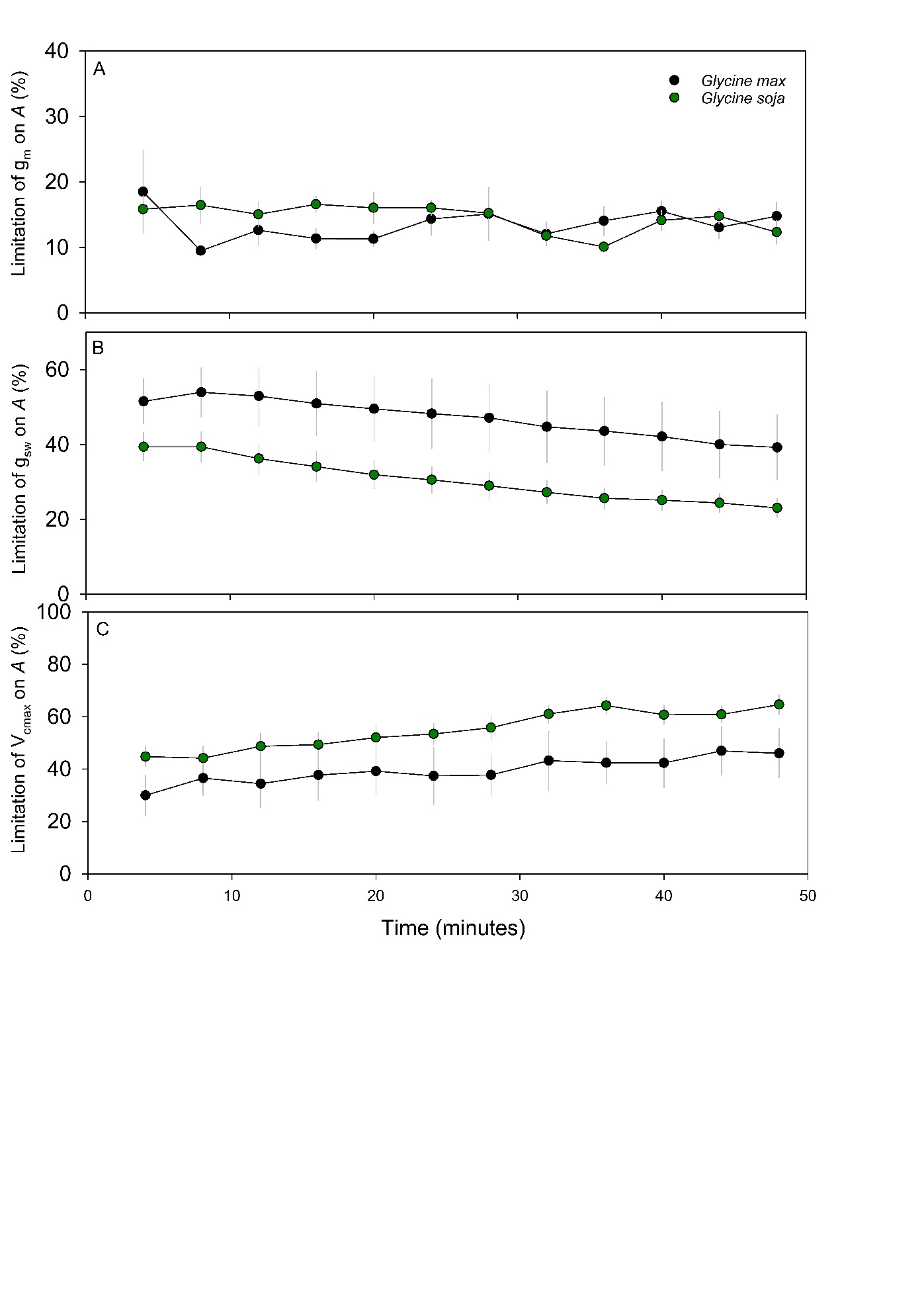
Supplementary Figure 8 – The limitation of mesophyll conductance (g_m_, A), stomatal conductance (g_sw,_, B) and maximum rate for the carboxylation of Ribulose 1,5-biphosphate ([RuBP] V_cmax_, C) on net CO_2_ assimilation rate (*A*) after an increase in light intensity over time assuming the biochemical limitation of RuBP carboxylation**.** Limitation calculations were made using the methods described in Grassi & Magnani, 2005. The comparisons are between domesticated high-yielding elite LD11 (*Glycine max* (L.) Merr, n = 4) and the average of four ancestor accessions (*Glycine soja* Siebold & Zucc, n = 16). Error bars indicate standard error.

Supplementary Figure 9 – The average temporal response of mesophyll conductance (*g_m_*) of high-yielding elite LD11 (*Glycine max* (L.) Merr) after a transition in photosynthetic photon flux density (PPFD) from 100 (grey area) to 1800 (white area). The comparisons are between *g_m_* calculated using the equations in Evans and Von Caemmerer, 2013 and Busch et al, 2020. Data points represent 4-minute moving averages.

**Supplementary methods**

The Grassi & Magnani (2005) framework defines limiting factors due to stomatal conductance, mesophyll conductance, and biochemistry, which we refer to here as $l_{s}^{Grassi}$, $l_{m}^{Grassi}$, and $l_{b}^{Grassi}$:

$l_{s}^{Grassi}=\frac{\frac{g_{tot}}{g_{sc}}\cdot\frac{\partial A}{\partial C_{c}}}{g_{tot}+\frac{\partial A}{\partial C_{c}}}$ Equation 1

$l_{m}^{Grassi}=\frac{\frac{g_{tot}}{g_{m}}\cdot\frac{\partial A}{\partial C_{c}}}{g_{tot}+\frac{\partial A}{\partial C_{c}}}$ Equation 2

$l_{b}^{Grassi}=\frac{g_{tot}}{g_{tot}+\frac{\partial A}{\partial C_{c}}}$ Equation 3

where $g_{tot}$ is the total conductance across the stomata and mesophyll, defined by

$\frac{1}{g_{tot}}=\frac{1}{g_{sc}}+\frac{1}{g_{m}}$ Equation 4

and $\frac{\partial A}{\partial C_{c}}$ is the partial derivative of the assimilation rate with respect to $C_{c}$, which can be calculated under Rubisco-limited assimilation from Equation 15 as

$\frac{\partial A_{c}}{\partial C_{c}}=\frac{V_{cmax}\cdot\left( \Gamma^{*}+K_{M} \right)}{\left( C_{c}+K_{M} \right)^{2}}$ Equation 5

or under RuBP-regeneration-limited assimilation from Equation 16 as

$\frac{\partial A_{j}}{\partial C_{c}}=\frac{J\cdot\Gamma^{*}\cdot\left( 4+8 \right)}{\left( {4\cdot C}_{c}+8\cdot\Gamma^{*} \right)^{2}}$ Equation 6
